# Supplementary material for: A systematic review and multivariate meta-analysis of the physical and mental health benefits of touch interventions
Source: Nat Hum Behav. 2024 Apr 8;8(6):1088–107. doi: 10.1038/s41562-024-01841-8 (PMC11199149; doi:10.1038/s41562-024-01841-8)
Supplement: Supplementary file 1 — Supplementary Figs. 1–21 and Tables 1–4. [file 41562_2024_1841_MOESM1_ESM.pdf]

nature human behaviour

Article https://doi.org/10.1038/s41562-024-01841-8

A systematic review and multivariate meta-analysis of the physical and mental health benefits of touch interventions

In the format provided by the authors and unedited

Supplementary Information

Supplementary Figures

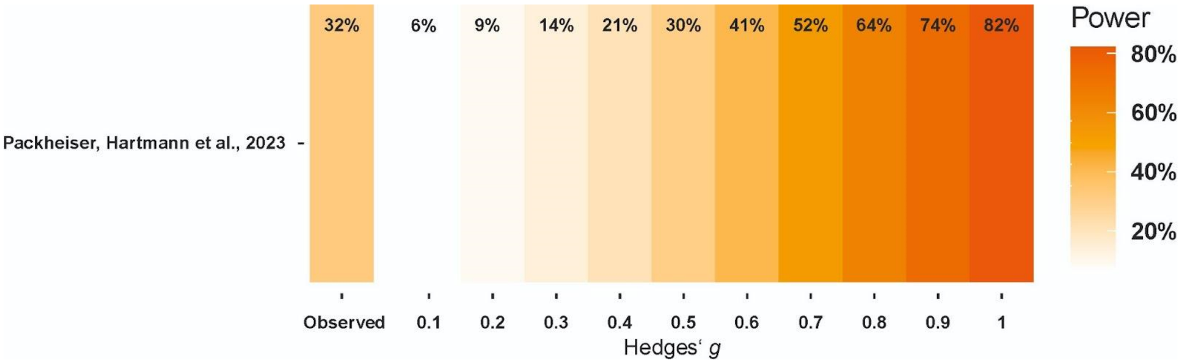

Supplementary Figure 1. Firepower plot (Quintana, 2023) for the meta-analysis of children/adults studies. The median power of each individual study in this meta-analysis was 32% to detect effect sizes of g = 0.52 suggesting that most studies were underpowered to reliably detect medium-sized effects.

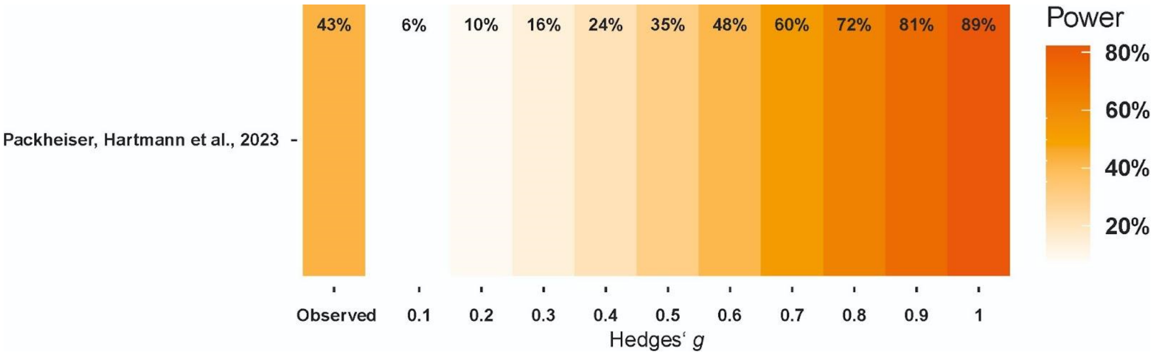

Supplementary Figure 2. Firepower plot (Quintana, 2023) for the meta-analysis of newborns studies. The median power of each individual study in this meta-analysis was 43% to detect effect sizes of g = 0.54 suggesting that most studies were underpowered to reliably detect medium-sized effects.

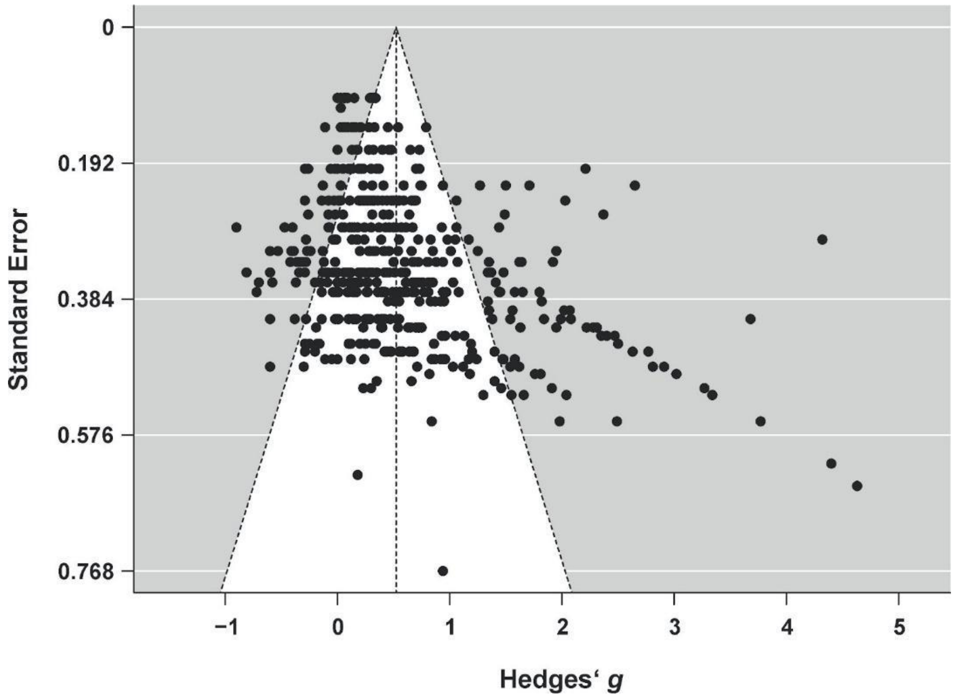

Supplementary Figure 3. Funnel plot for the adult meta-analysis. Small study bias is indicated by funnel plot asymmetry.

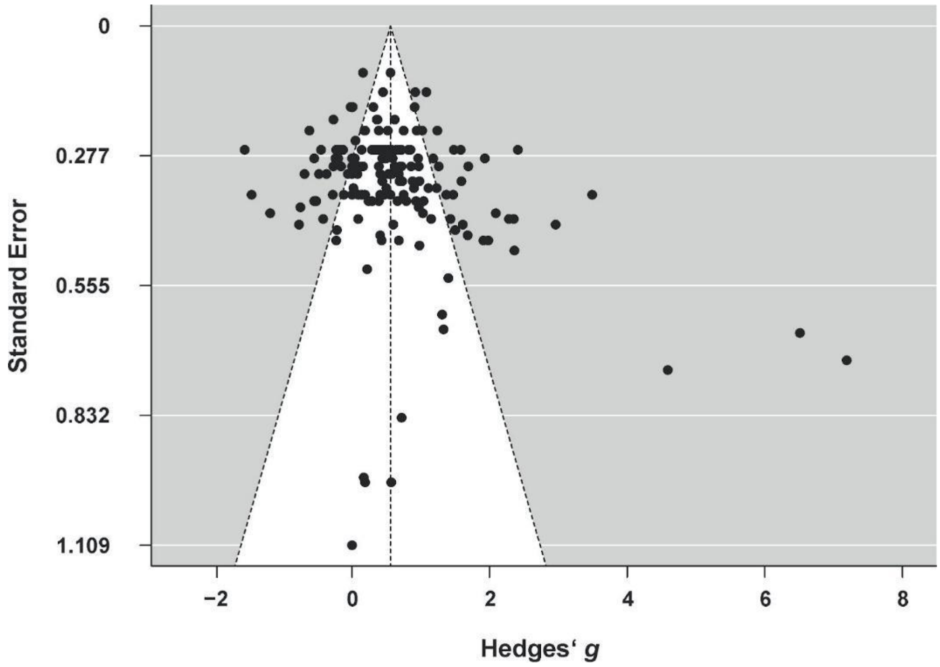

Supplementary Figure 4. Funnel plot for the meta-analysis of newborns studies. Small study bias is indicated by funnel plot asymmetry.

-

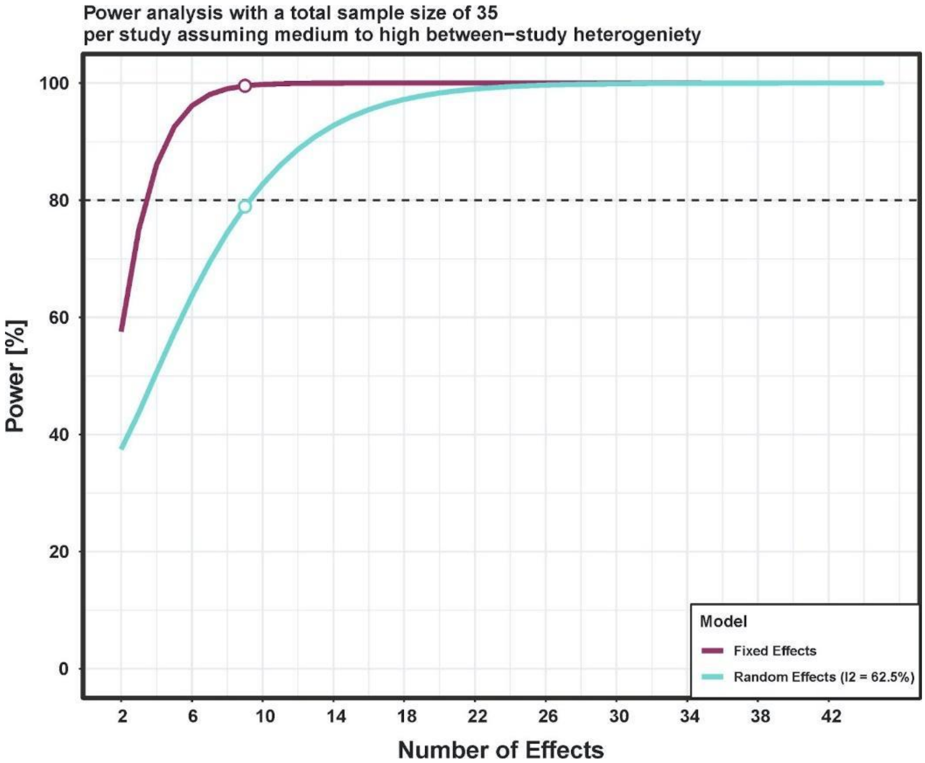

Supplementary Figure 5. Power calculations for the meta-analysis of children/adults studies using the metapower package (Griffin, 2021). Random effects models reached 80% power assuming medium to high heterogeneity at around nine effects.

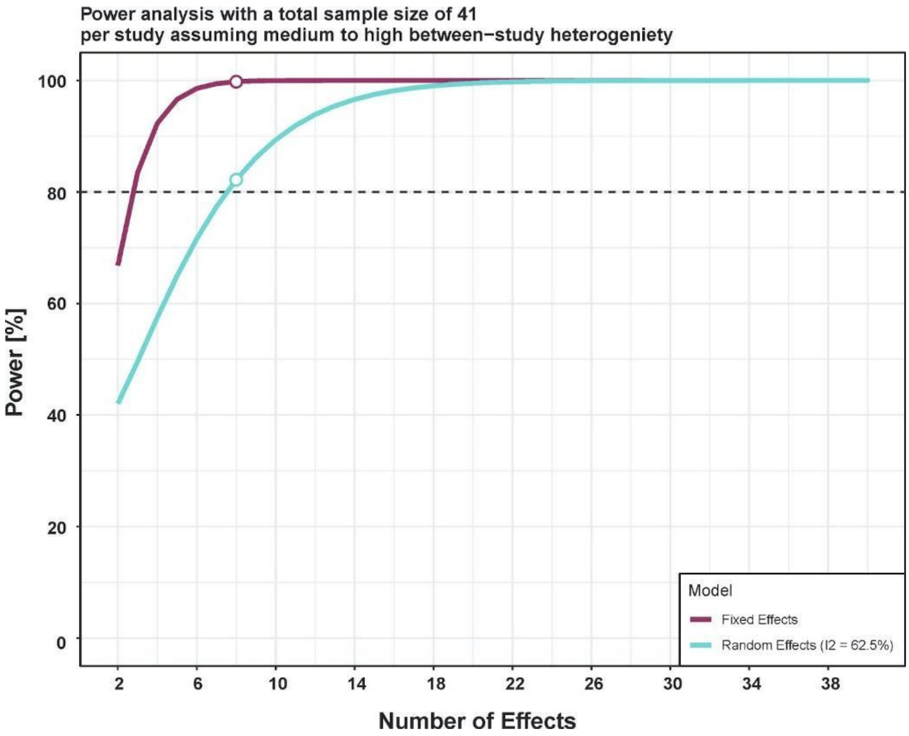

Supplementary Figure 6. Power calculations for the meta-analysis of newborns studies using the metapower package (Griffin, 2021). Random effects models reached 80% power assuming medium to high heterogeneity at around nine effects.

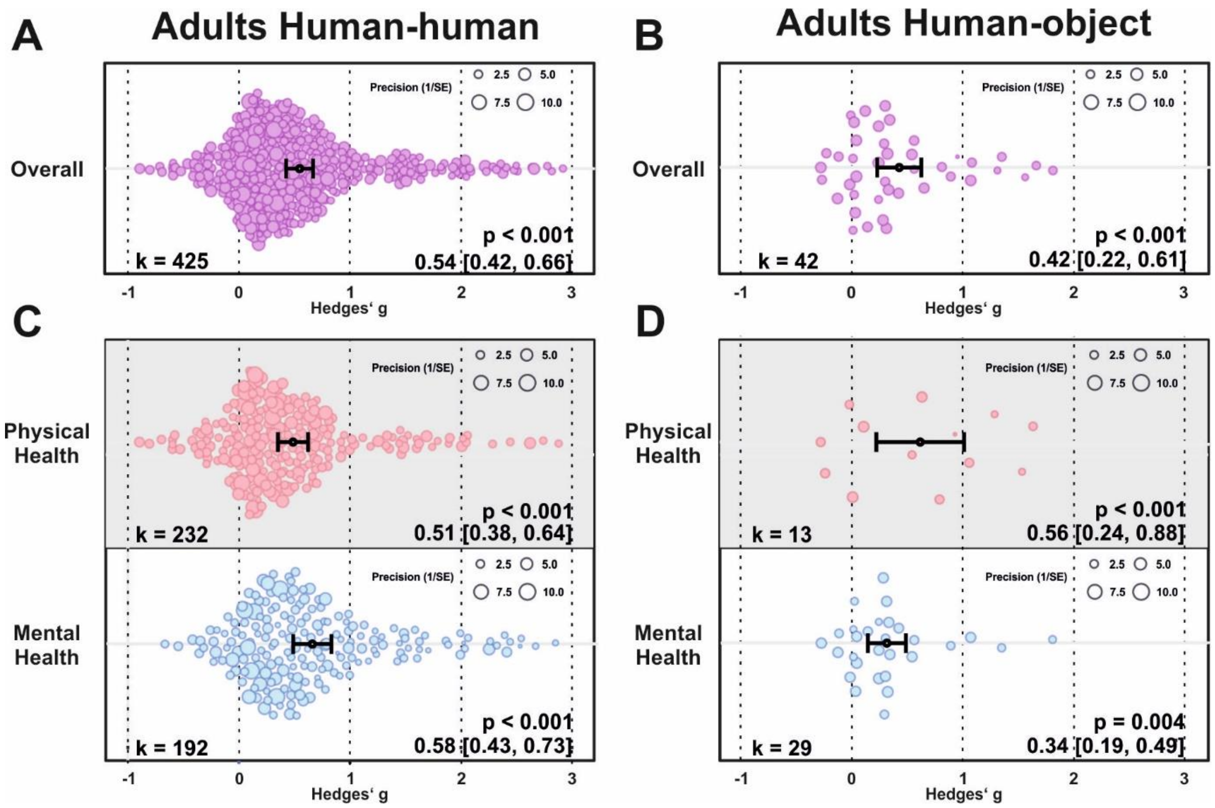

Supplementary Figure 7. (A) Orchard plot illustrating the overall benefits across all health outcomes for human-human (A) and human-object touch (B) for 467 in part dependent effect sizes from 85 studies and 101 cohorts. (C-D) same as (A-B) but separating the results for physical vs mental health benefits across 467 in part dependent effect sizes from 85 studies and 101 cohorts. Each dot reflects a measured effect and the number of effects (k) included in the analysis is depicted in the bottom left. Mean effects and 95% CIs are presented in the bottom right. Overall effects of moderator impact were assessed via an F-test and post hoc comparisons were done using t-tests (two-sided test). Note that the p-values above the mean effects indicate whether an effect differed significantly from a zero effect. P-values were not corrected for multiple comparisons. Dot size reflects precision of each individual effect (larger = higher precision).

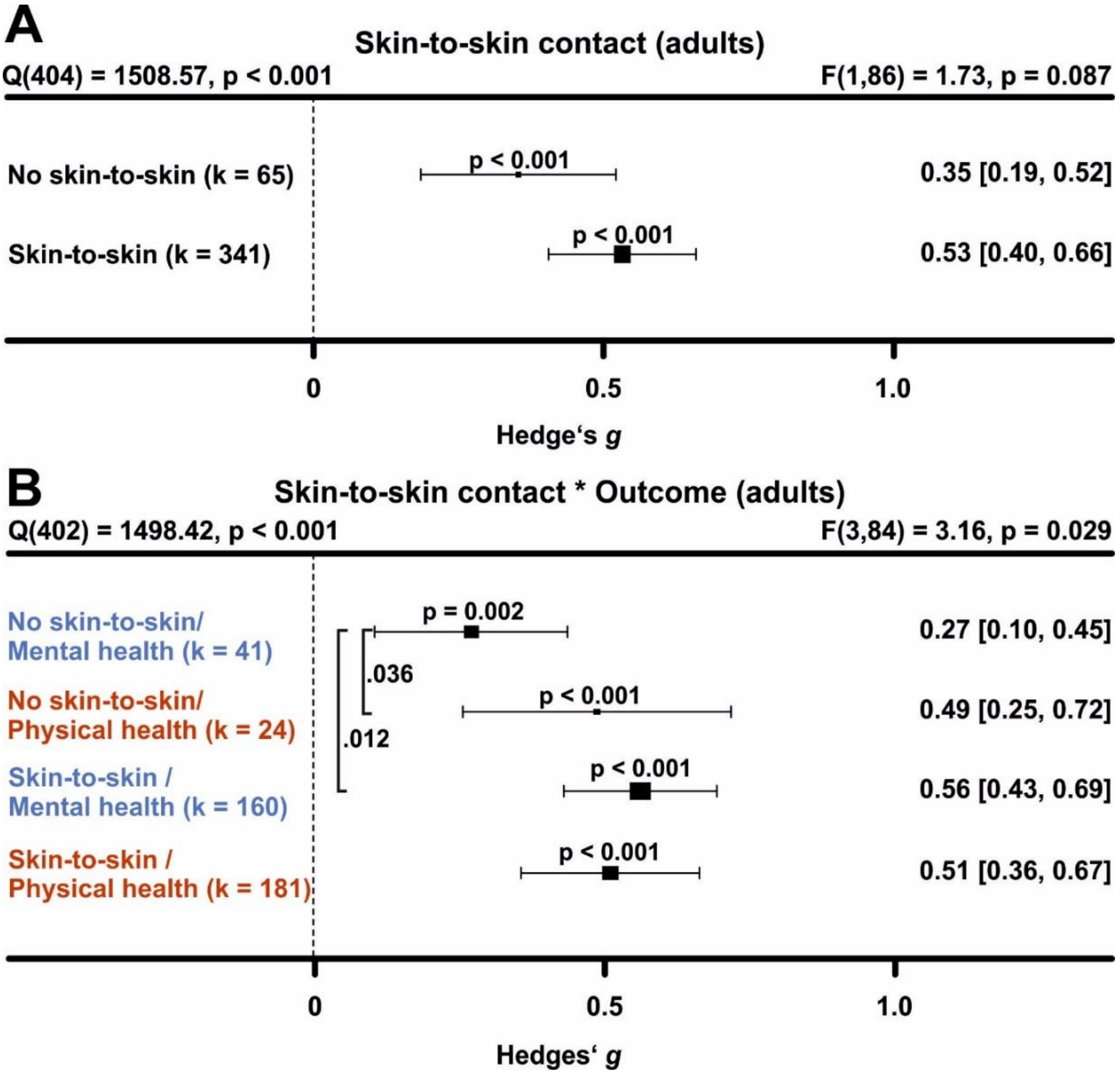

Supplementary Figure 8. Comparing health benefits of skin-to-skin contact during touch. (A) Health benefits for present and absent skin-to-skin contact. 406 in part dependent effect sizes from 71 studies and 88 cohorts were included in this analysis. The analysis includes human-human, human-object and human-animal studies. All studies without human-human interactions were classified as absent skin-to-skin contact. (B) Same as A, but separating mental vs physical health benefits across 406 in part dependent effect sizes from 71 studies and 88 cohorts. The number of effects per moderator included in the analysis are indicated on the left (k). Numbers on the right represent the mean effect, its 95% CI in square brackets and the significance level estimating the likelihood that the effect is equal to zero. Overall effects of moderator impact were assessed via an F-test and post hoc comparisons were done using t-tests (two-sided test). The F-value in the top right represents a test of the hypothesis that all effects within the subpanel are equal. The Q statistic represents heterogeneity. P-values of post hoc tests are depicted whenever significant. Note that the p-values above the horizontal whiskers indicate whether an effect differed significantly from a zero effect whereas the vertical lines indicate significant post hoc tests between moderator levels. P-values were not corrected for multiple comparisons. Physical outcomes are marked in red, mental outcomes are marked in blue.

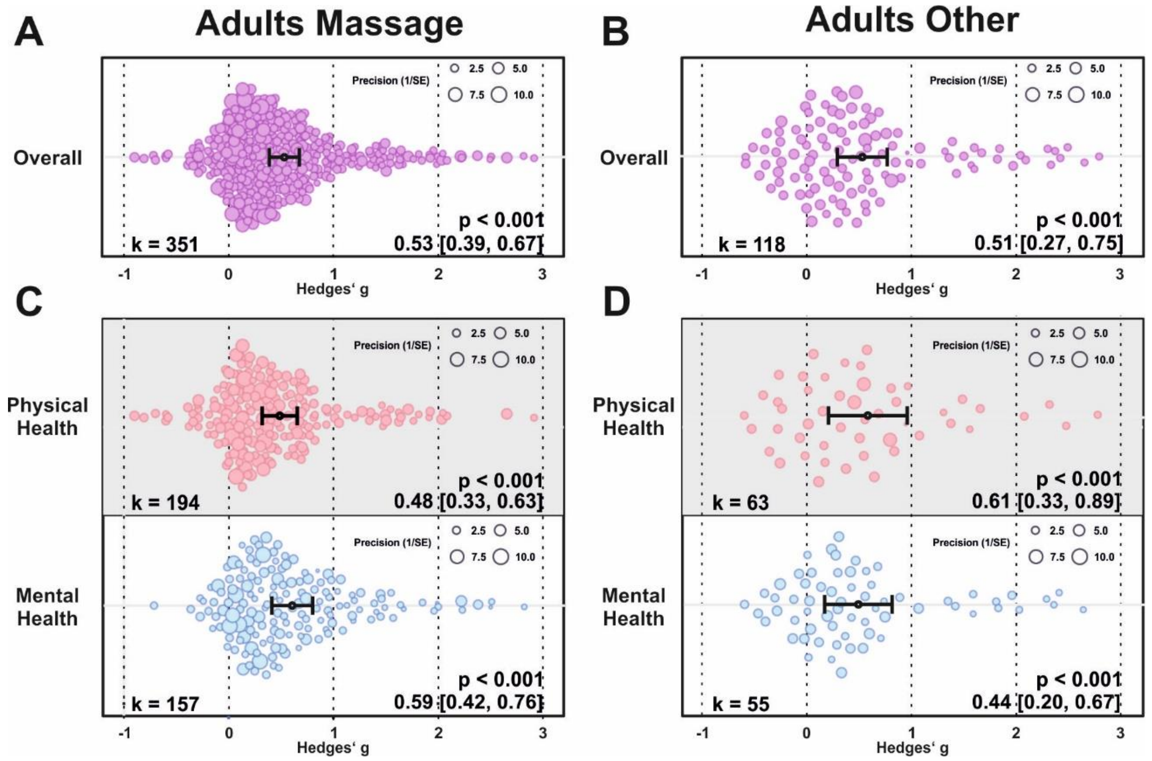

Supplementary Figure 9. Orchard plot illustrating the overall benefits across all health outcomes for massage interventions (A) and other types of touch interventions in adult cohorts (B) across 469 in part dependent effect sizes from 85 studies and 103 cohorts. (C-D) same as (A-B) but separating the results for physical vs mental health benefits across 469 in part dependent effect sizes from 85 studies and 103 cohorts. Each dot reflects a measured effect and the number of effects (k) included in the analysis is depicted in the bottom left. Mean effects and 95% CIs are presented in the bottom right. Overall effects of moderator impact were assessed via an F-test and post hoc comparisons were done using t-tests (two-sided test). Note that the p-values above indicate whether an effect differed significantly from a zero effect. P-values were not corrected for multiple comparisons. Dot size reflects precision of each individual effect (larger = higher precision).

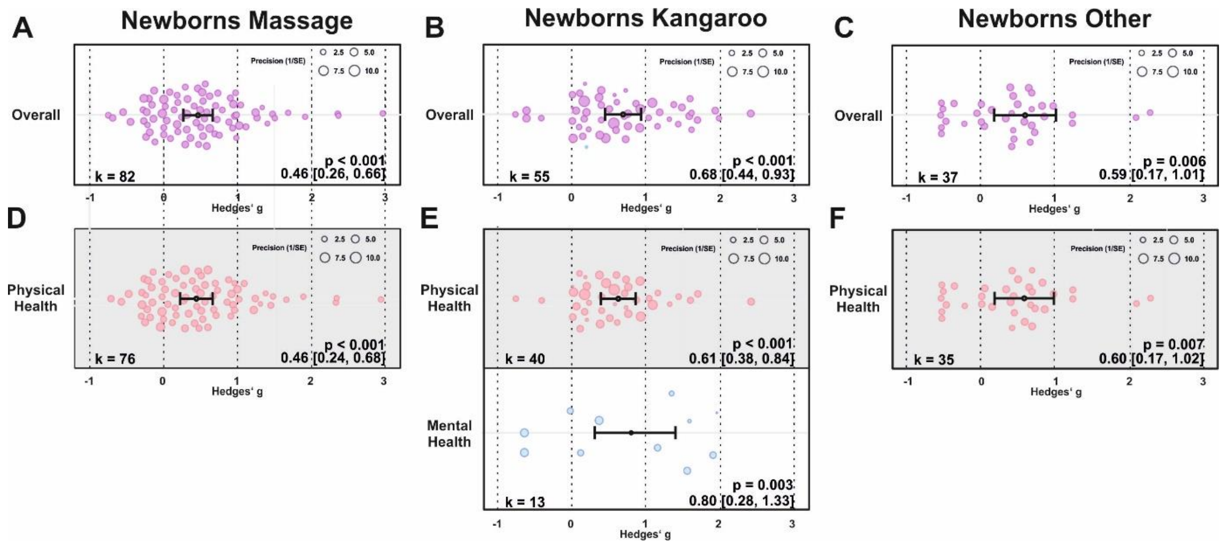

Supplementary Figure 10. (A) Orchard plot illustrating the overall benefits across all health outcomes for massage interventions (A), the effects of kangaroo care (B) and other types of touch interventions (C) in newborns across 174 in part dependent effect sizes from 52 studies and 63 cohorts. (E-F) same as (A-C) but separating the results for physical vs mental health benefits if sufficient effects for further analysis could be found. In total, 164 in part dependent effect sizes from 51 studies and 62 cohorts were analyzed. Each dot reflects a measured effect and the number of effects (k) included in the analysis is depicted in the bottom left. Mean effects and 95% CIs are

presented in the bottom right. Overall effects of moderator impact were assessed via an F-test and post hoc comparisons were done using t-tests (two-sided test). Note that the p-values above indicate whether an effect differed significantly from a zero effect. P-values were not corrected for multiple comparisons. Dot size reflects precision of each individual effect (larger = higher precision).

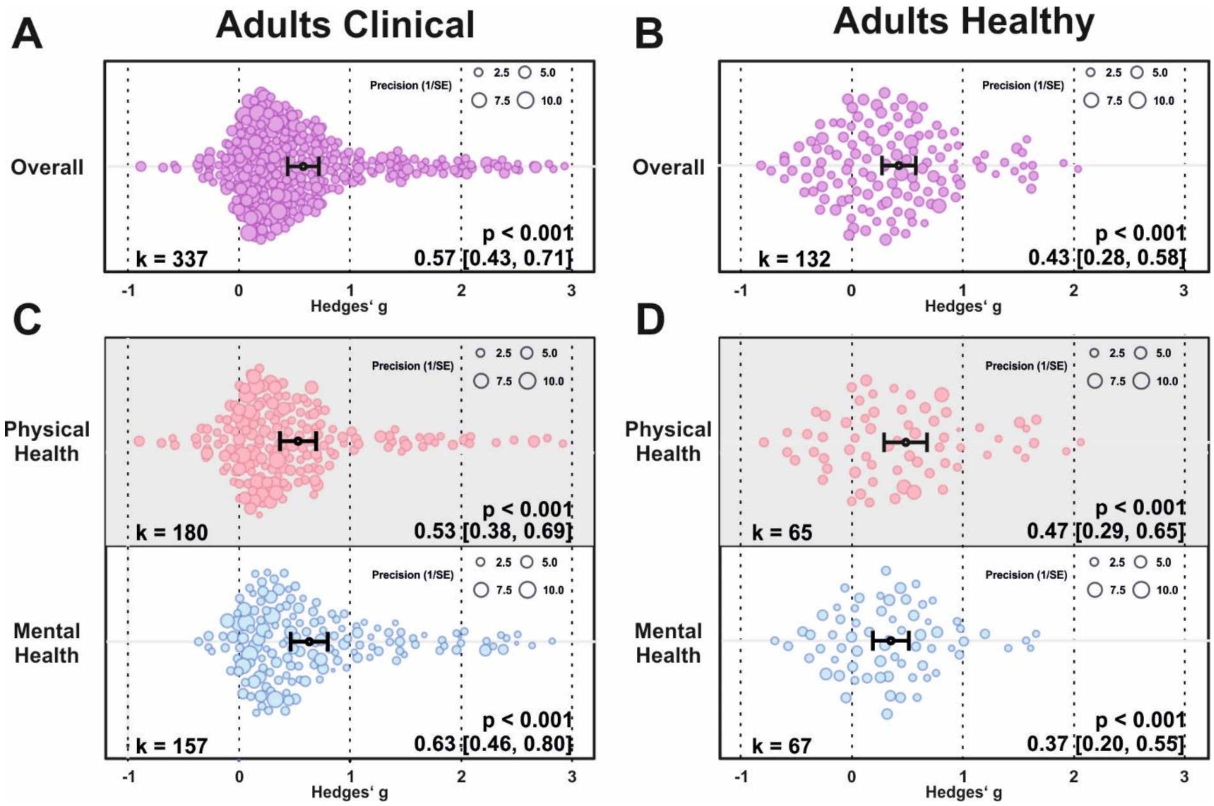

Supplementary Figure 11. (A) Orchard plot illustrating the overall benefits across all health outcomes for clinical (A) and healthy adult cohorts (B) across 469 in part dependent effect sizes from 85 studies and 103 cohorts. (C-D) same as (A-B) but separating the results for physical vs mental health benefits across 469 in part dependent effect sizes from 85 studies and 103 cohorts. Each dot reflects a measured effect and the number of effects (k) included in the analysis is depicted in the bottom left. Mean effects and 95% CIs are presented in the bottom right. Overall effects of moderator impact were assessed via an F-test and post hoc comparisons were done using t-tests (two-sided test). Note that the p-values above indicate whether an effect differed significantly from a zero effect. P-values were not corrected for multiple comparisons. Dot size reflects precision of each individual effect (larger = higher precision).

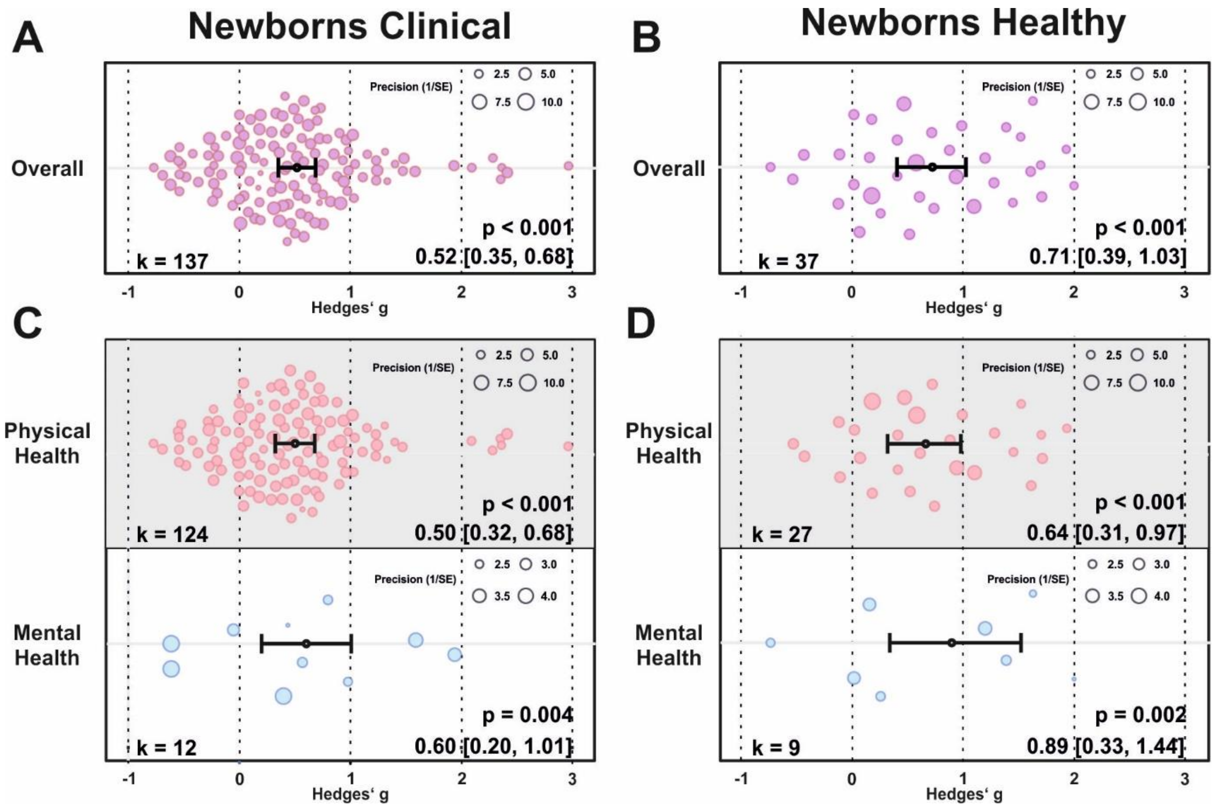

Supplementary Figure 12. (A) Orchard plot illustrating the overall benefits across all health outcomes for clinical (A) and healthy newborns (B) across 174 in part dependent effect sizes from 52 studies and 63 cohorts. (C-D) same as (A-B) but separating the results for physical vs mental health benefits across 172 in part dependent effect sizes from 52 studies and 63 cohorts. Each dot reflects a measured effect and the number of effects (k) included in the analysis is depicted in the bottom left. Mean effects and 95% CIs are presented in the bottom right. Overall effects of moderator impact were assessed via an F-test and post hoc comparisons were done using t-tests (two-sided test). Note that the p-values above indicate whether an effect differed significantly from a zero effect. P-values were not corrected for multiple comparisons. Dot size reflects precision of each individual effect (larger = higher precision).

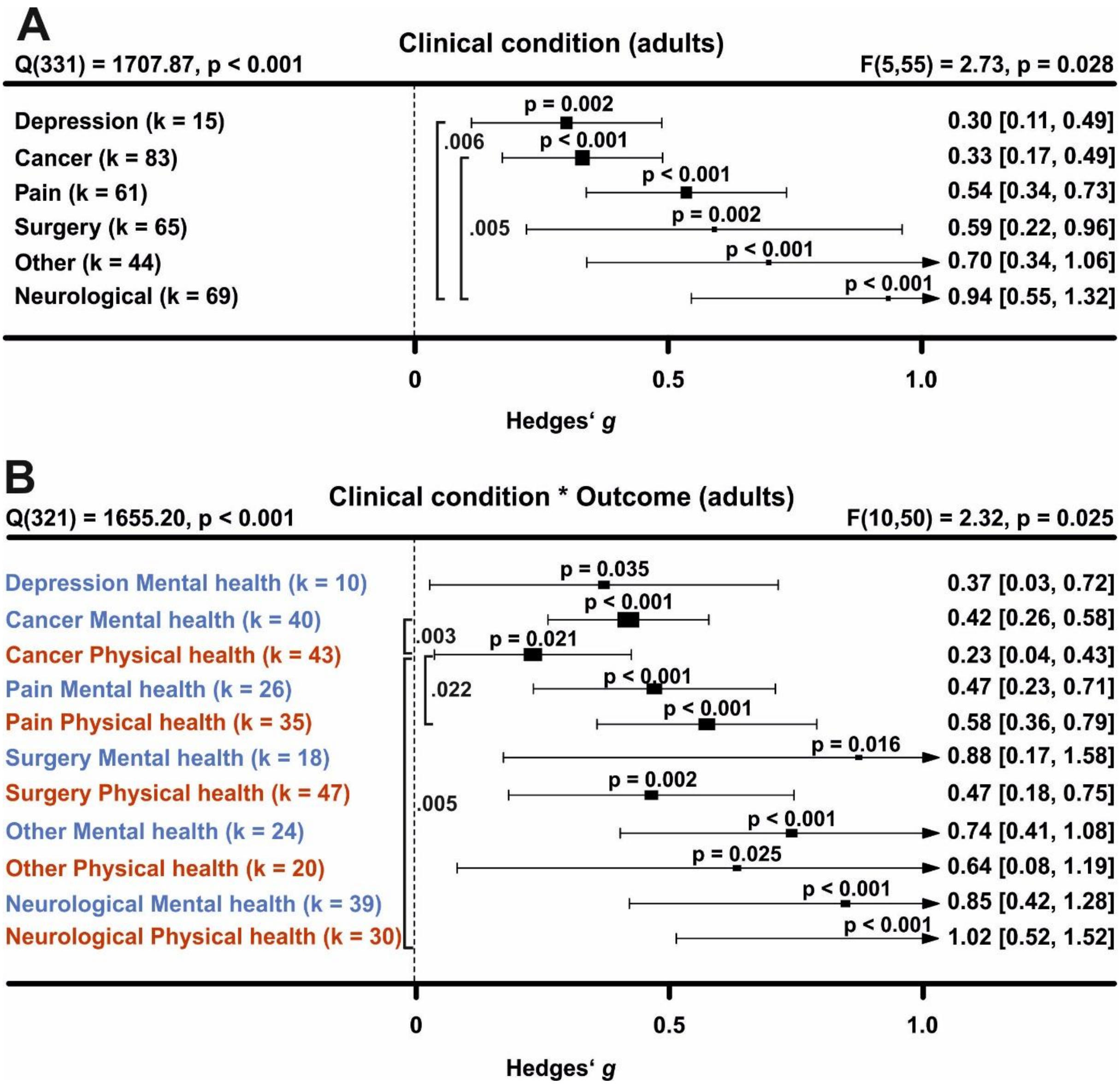

Supplementary Figure 13. Comparing health benefits across different clinical disorders. (A) Health benefits in individuals diagnosed with depression, cancer, pain syndromes, following surgery, neurological or other types of disorders across 337 in part dependent effect sizes from 56 studies and 61 cohorts. (B) Same as A, but separating mental vs physical health benefits across 332 in part dependent effect sizes from 56 studies and 61 cohorts. The number of effects per moderator included in the analysis are indicated on the left (k). Numbers on the right represent the mean effect, its 95% CI in square brackets and the significance level estimating the likelihood that the effect is equal to zero. Overall effects of moderator impact were assessed via an F-test and post hoc comparisons were done using t-tests (two-sided test). The F-value in the top right represents a test of the hypothesis that all effects within the subpanel are equal. The Q statistic represents heterogeneity. P-values of post hoc tests are depicted whenever significant. Note that the p-values above the horizontal whiskers indicate whether an effect differed significantly from a zero effect whereas the vertical lines indicate significant post hoc tests between moderator levels. P-values were not corrected for multiple comparisons. Physical outcomes are marked in red, mental outcomes are marked in blue.

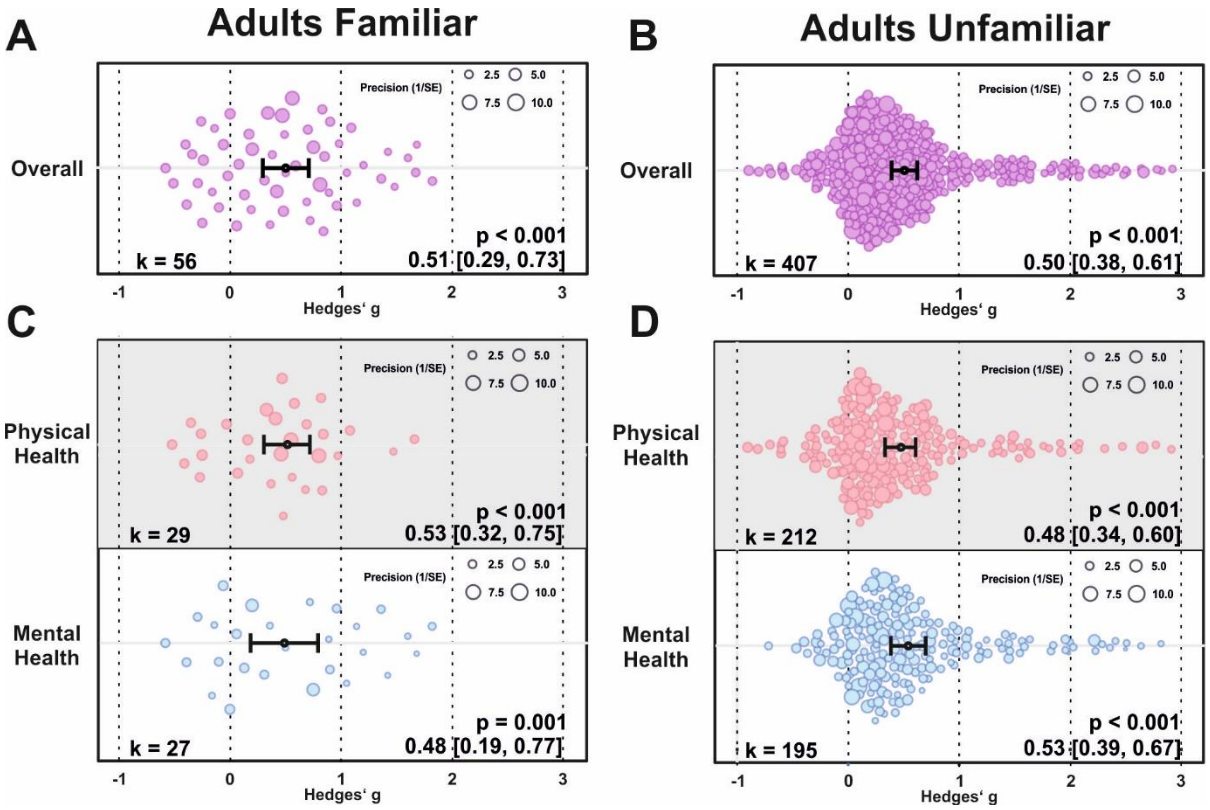

Supplementary Figure 14. (A) Orchard plot illustrating the overall benefits across all health outcomes for touch applied by a familiar (A) and unfamiliar individual in adult cohorts (B) across 463 in part dependent effect sizes from 83 studies and 101 cohorts. (C-D) same as (A-B) but separating the results for physical vs mental health benefits across 463 in part dependent effect sizes from 83 studies and 101 cohorts. Each dot reflects a measured effect and the number of effects (k) included in the analysis is depicted in the bottom left. Mean effects and 95% CIs are presented in the bottom right. Overall effects of moderator impact were assessed via an F-test and post hoc comparisons were done using t-tests (two-sided test). Note that the p-values above indicate whether an effect differed significantly from a zero effect. P-values were not corrected for multiple comparisons. Dot size reflects precision of each individual effect (larger = higher precision).

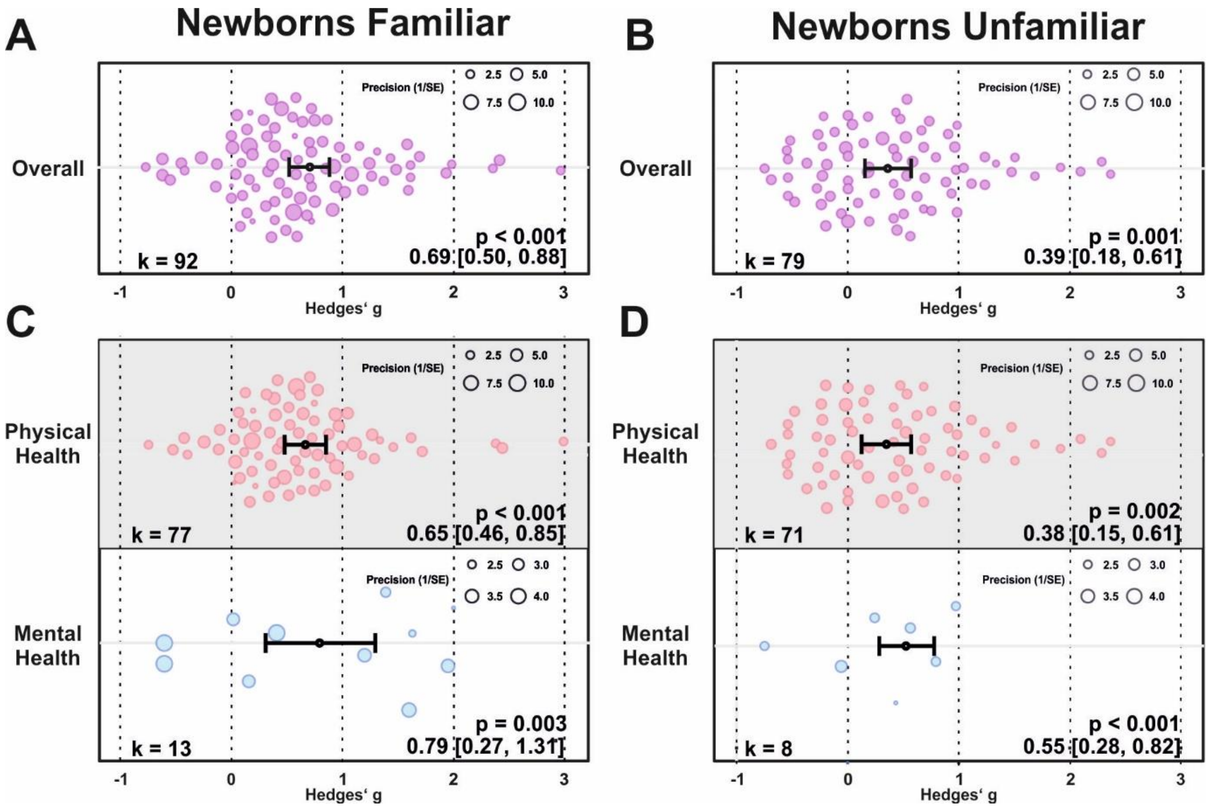

Supplementary Figure 15. (A) Orchard plot illustrating the overall benefits across all health outcomes for touch applied by a familiar (A) and unfamiliar individual in newborns (B) across 171 in part dependent effect sizes from 51 studies and 62 cohorts. (C-D) same as (A-B) but separating the results for physical vs mental health benefits across 169 in part dependent effect sizes from 51 studies and 62 cohorts. Each dot reflects a measured effect and the number of effects (k) included in the analysis is depicted in the bottom left. Mean effects and 95% CIs are presented in the bottom right. Overall effects of moderator impact were assessed via an F-test and post hoc comparisons were done using t-tests (two-sided test). Note that the p-values above indicate whether an effect differed significantly from a zero effect. P-values were not corrected for multiple comparisons. Dot size reflects precision of each individual effect (larger = higher precision).

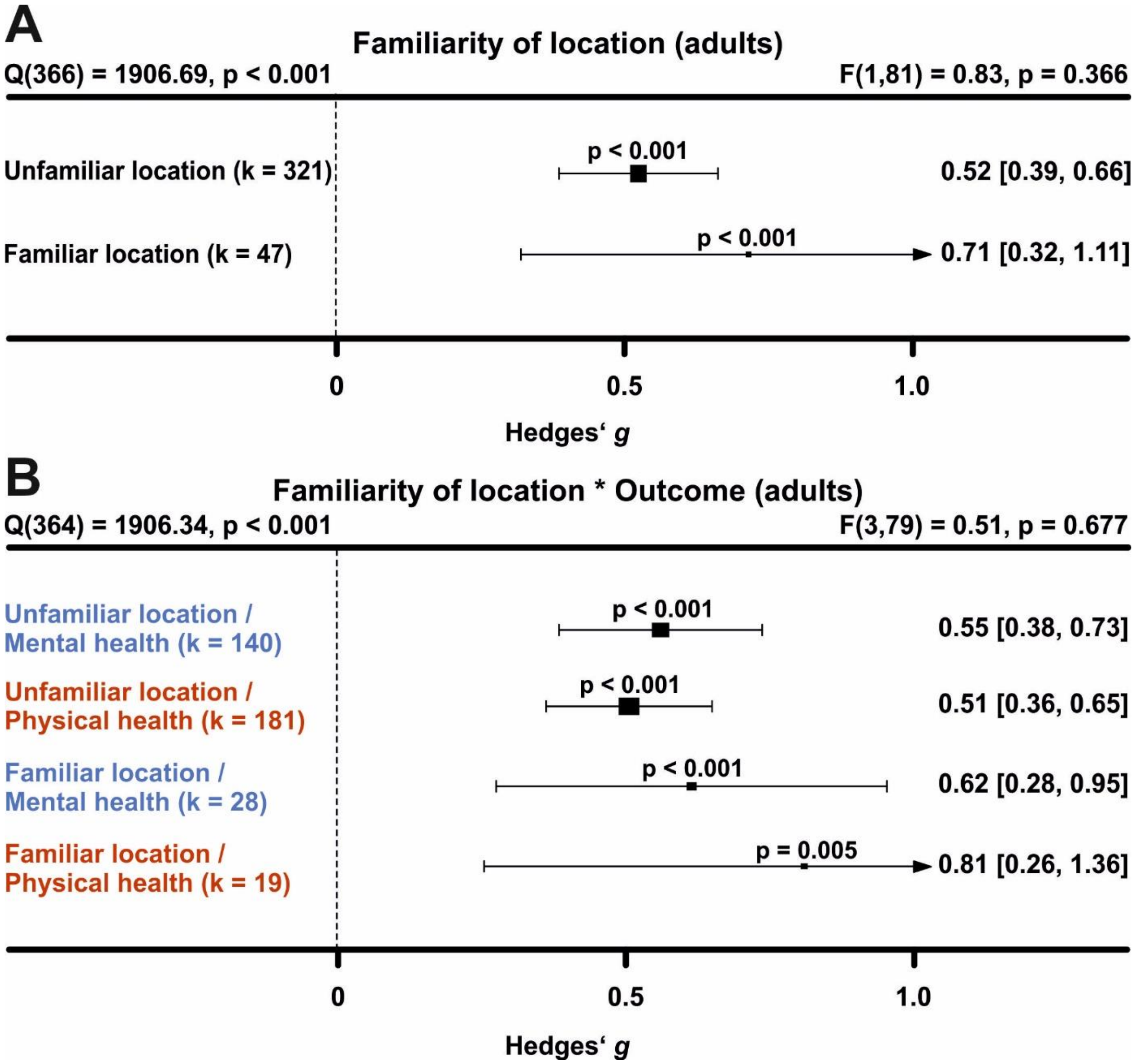

Supplementary Figure 16. Comparing health benefits depending on familiarity with the intervention location. (A) Health benefits separated by intervention location in adults (A) and newborns (B) across 368 in part dependent effect sizes from 68 studies and 83 cohorts. (C-D) Same as (A-B), but separating mental vs physical health benefits across 368 in part dependent effect sizes from 68 studies and 83 cohorts. The number of effects per moderator included in the analysis are indicated on the left (k). Numbers on the right represent the mean effect, its 95% CI in square brackets and the significance level estimating the likelihood that the effect is equal to zero. Overall effects of moderator impact were assessed via an F-test and post hoc comparisons were done using t-tests (two-sided test). The F-value in the top right represents a test of the hypothesis that all effects within the subpanel are equal. The Q statistic represents heterogeneity. P-values of post hoc tests are depicted whenever significant. Note that the p-values above the horizontal whiskers indicate whether an effect differed significantly from a zero effect whereas the vertical lines indicate significant post hoc tests between moderator levels. P-values were not corrected for multiple comparisons. Physical outcomes are marked in red, mental outcomes are marked in blue.

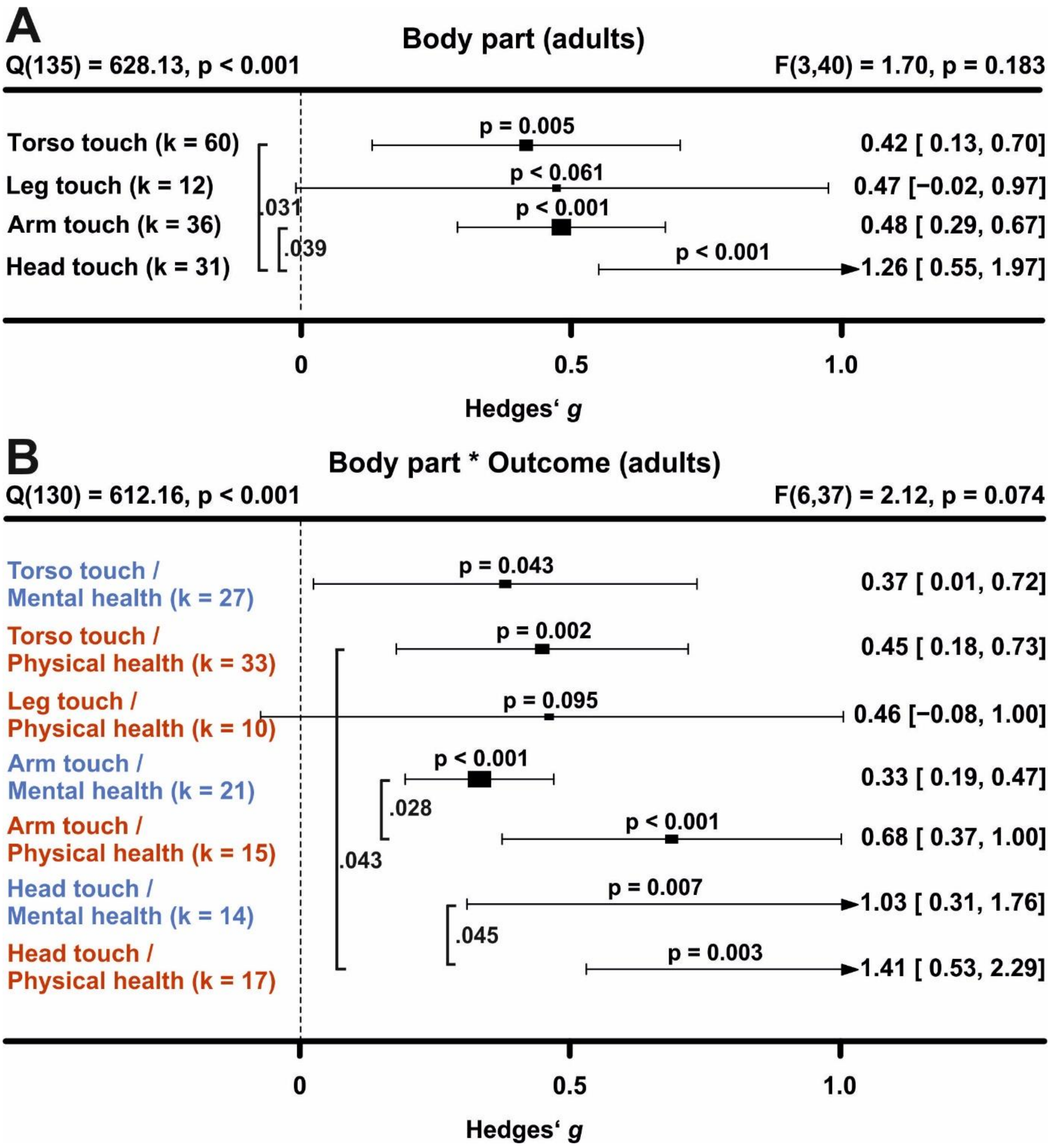

Supplementary Figure 17. Comparing health benefits depending on the touched body part. (A) Health benefits depend on the location where touch was applied (arm, leg, torso or head). Studies in which multiple body parts were touched were excluded from the analysis across 139 in part dependent effect sizes from 33 studies and 44 cohorts. (B) Same as A, but separating mental vs physical health benefits across 137 in part dependent effect sizes from 33 studies and 44 cohorts. The number of effects per moderator included in the analysis are indicated on the left (k). Note that there was an insufficient number of effects for further analysis of mental health benefits following leg touch. Numbers on the right represent the mean effect, its 95% CI in square brackets and the significance level estimating the likelihood that the effect is equal to zero. Overall effects of moderator impact were assessed via an F-test and post hoc comparisons were done using t-tests (two-sided test). The F-value in the top right represents a test of the hypothesis that all effects within the subpanel are equal. The Q statistic represents heterogeneity. P-values of post hoc tests are depicted whenever significant. Note that the p-values above the horizontal whiskers indicate whether an effect differed significantly from a zero effect whereas the vertical lines indicate significant post hoc tests between moderator levels. P-values were not corrected for multiple comparisons. Physical outcomes are marked in red, mental outcomes are marked in blue.

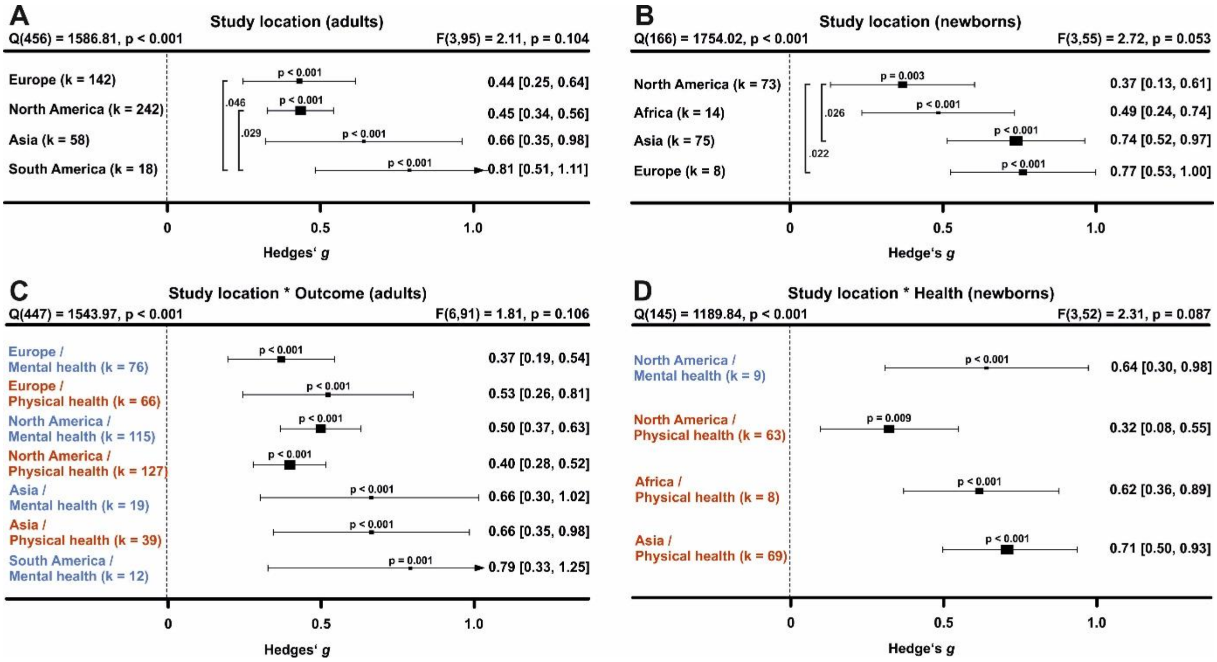

Supplementary Figure 18. Comparing health benefits depending on study location. (A) Health benefits separated by study location in adults (A) across 460 in part dependent effect sizes from 81 studies and 99 cohorts and newborns (B) across 170 in part dependent effect sizes from 49 studies and 59 cohorts. (C) Same as (A), but separating mental vs physical health benefits across 454 in part dependent effect sizes from 80 studies and 98 cohorts. (D) Same as (B), but separating mental vs physical health benefits across 149 in part dependent effect sizes from 46 studies and 56 cohorts. The number of effects per moderator included in the analysis are indicated on the left (k). Note that there was an insufficient number of effects for further analysis for certain regions both for the overall and the specific effects on physical or mental health in both meta-analyses. Numbers on the right represent the mean effect, its 95% CI in square brackets and the significance level estimating the likelihood that the effect is equal to zero. Overall effects of moderator impact were assessed via an F-test and post hoc comparisons were done using t-tests (two-sided test). The F-value in the top right represents a test of the hypothesis that all effects within the subpanel are equal. The Q statistic represents heterogeneity. P-values of post hoc tests are depicted whenever significant. Note that the p-values above the horizontal whiskers indicate whether an effect differed significantly from a zero effect whereas the vertical lines indicate significant post hoc tests between moderator levels. P-values were not corrected for multiple comparisons. Physical outcomes are marked in red, mental outcomes are marked in blue.

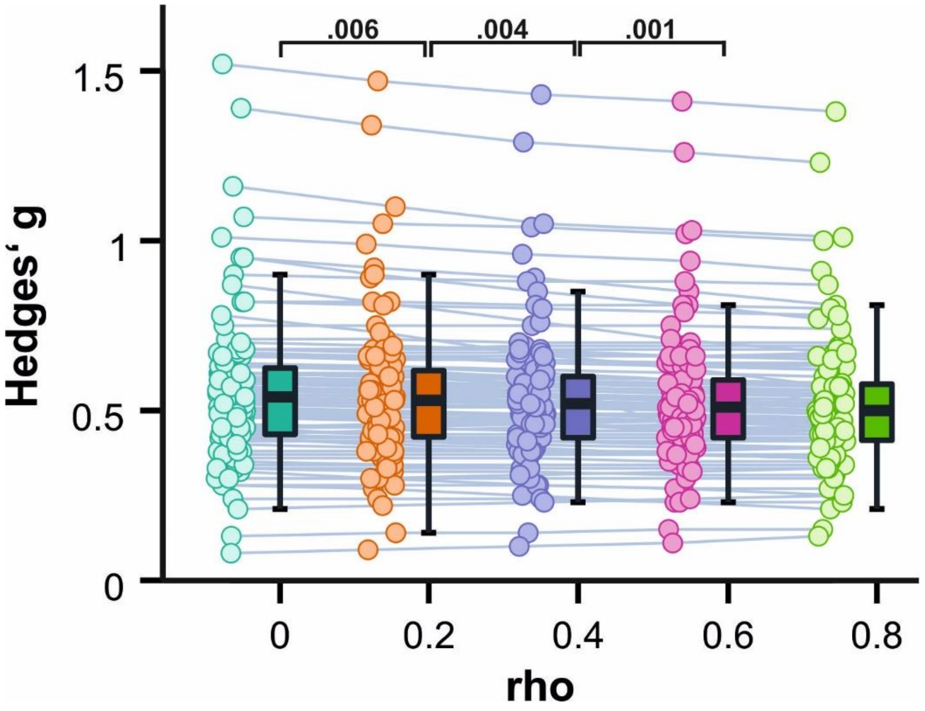

Supplementary Figure 19. Influence of different levels of rho across all 102 independently computed effect estimates for categorical moderators in the adults meta-analysis. P-values of post hoc tests are depicted whenever significant (corrected for multiple comparisons using the Bonferroni method). Assuming lower levels of correlation between effect sizes resulted in slightly higher effect estimates (repeated measures ANOVA, two-sided test). Thus, higher values of rho can be considered as conservative estimates. No significant difference was observed between high correlation values of rho at 0.6 (used value in the manuscript) and 0.8. Effect estimates and confidence intervals for each computed outcome variable can be found on OSF in the Supplementary Table "Sensitvity analyses". Boxplots reflect the median and the first and third quartile. Whiskers represent the minimum (first quartile * 1.5 interquartile range) and maximum (third quartile * 1.5 interquartile range).

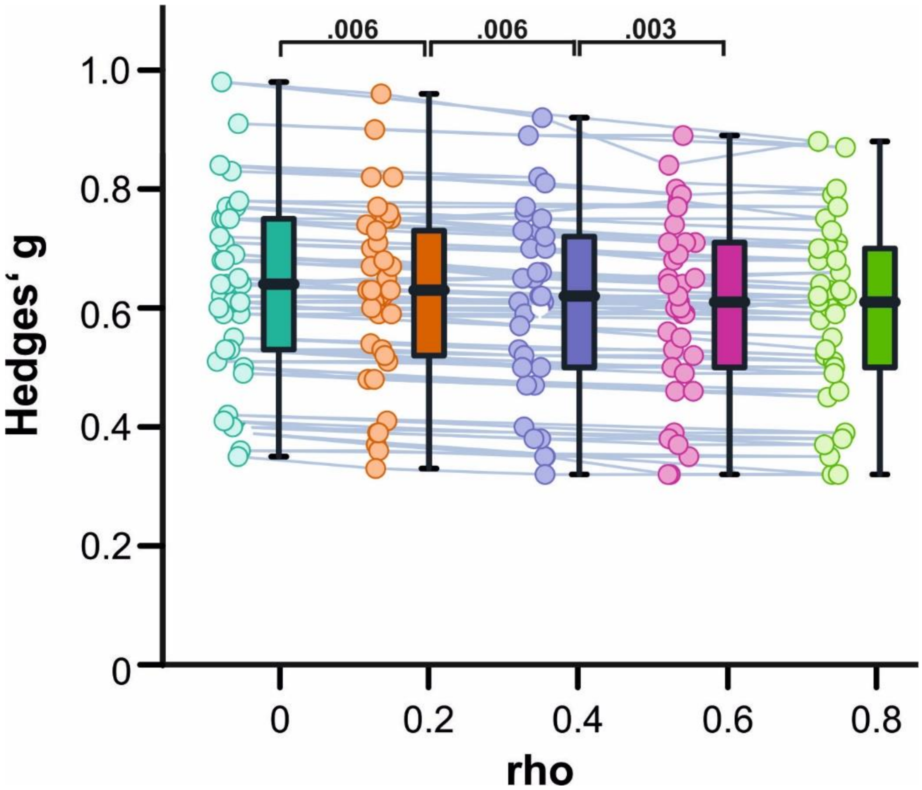

Supplementary Figure 20. Influence of different levels of rho across all 37 independently computed effect estimates for categorical moderators in the newborn meta-analysis. P-values of post hoc tests are depicted whenever significant (corrected for multiple comparisons using the Bonferroni method). Identically to the results for the adults meta-analysis, assuming lower levels of correlation between effect sizes resulted in slightly higher effect estimates (repeated measures ANOVA, two-sided test). No significant difference was observed between high correlation values of rho at 0.6 (used value in the manuscript) and 0.8. Effect estimates and confidence intervals for each computed outcome variable can be found on OSF in the Supplementary Table "Sensitvity analyses". Boxplots reflect the median and the first and third quartile. Whiskers represent the minimum (first quartile * 1.5 interquartile range) and maximum (third quartile * 1.5 interquartile range).

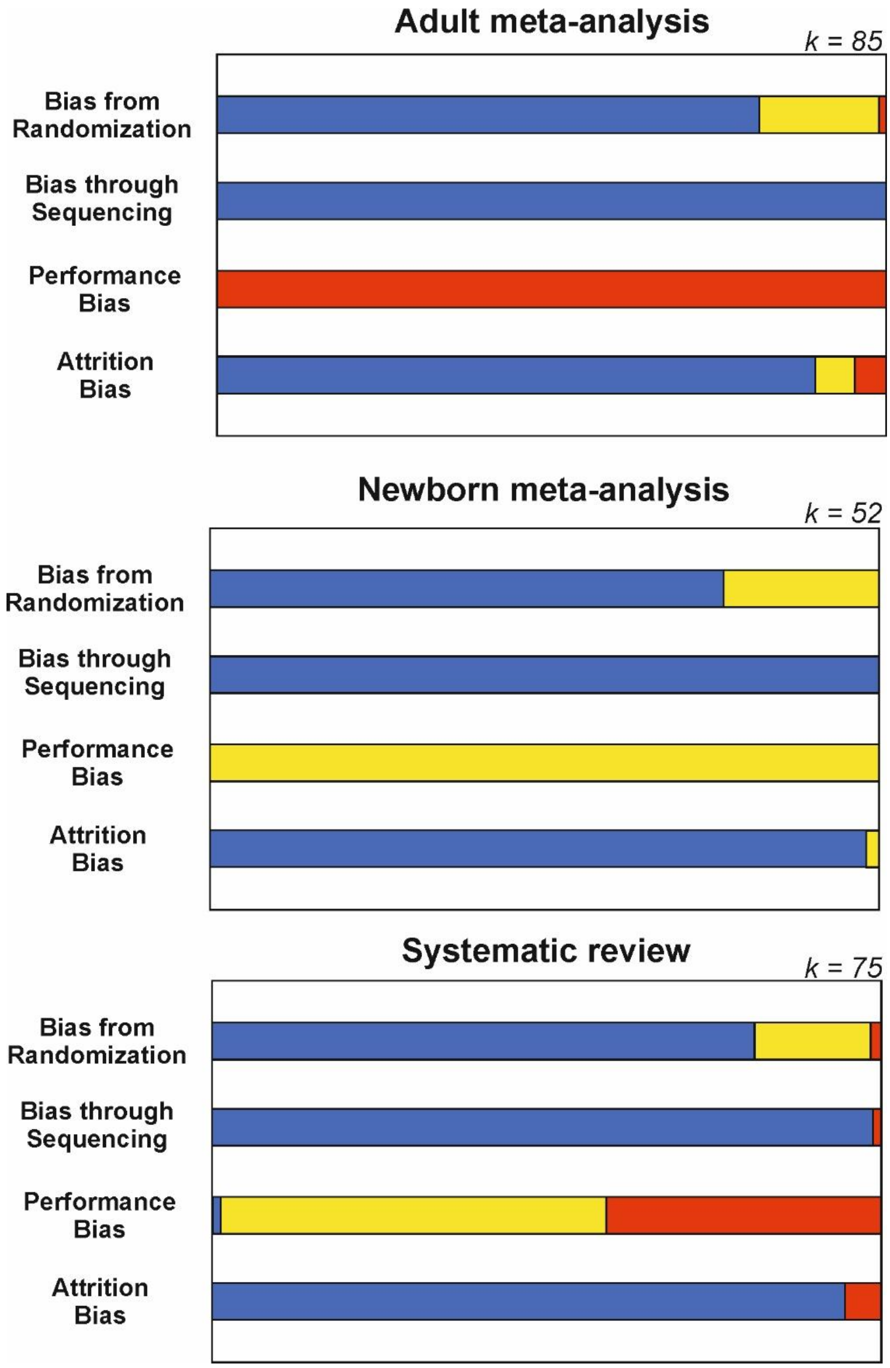

Supplementary Figure 21. Risk of bias overview. Results from the assessment of randomization, sequence, performance and attrition bias. Bias from randomization included whether a randomization was performed and whether there were baseline differences. If one of these variables was present, the risk was assessed as medium and if both were present, the risk was assessed as high. Sequence bias was low if the order of conditions were randomized. Note that sequence bias was only possible in within-subjects cross-over designs that were excluded from both meta-analyses. Performance bias was classified as high if both the participant and experimenter were aware of the experimental condition and classified as medium if only one group was aware. Performance bias was always high in the adult meta-analysis as blinding of the participants and experimenters to the experimental conditions was not possible due to the nature of the intervention. For studies with newborns and animals, we assessed the performance bias as medium since neither newborns nor animals were aware of being part of an experimental group. Attrition bias was classified as low if there was no attrition or it was identical across experimental and control group. Medium attrition bias was assessed if the difference was between up to five individuals between experimental and control group. High attrition bias constituted anything beyond that. Blue = Low risk of bias, Yellow = Medium risk of bias, Red = High risk of bias. Bar length corresponds to percentages.

Supplementary Tables

Supplementary Table 1. List of touch interventions of touched body parts across the included studies

List of touch interventions	List of touch body parts
Massage therapy (including self-massage)	Head (including face)
Kangaroo care (including skin-to-skin contact) for newborns only	Arm (including hands)
	Leg (including feet)
Other touch (including gentle touch, Yakson touch, maternal touch, tactile and kinesthetic stimulation, haptic touch, vibration touch, superficial touch, object touch, physical touch, self touch, stroking, rocking, fondling, petting, patting, acupressure, hugging, close contact, gentle touch, passive movement, child touch, kangaroo (for adults/children only))	Torso (including back, chest, stomach and neck)
	Multi (whenever multiple parts of the body were touched, e.g. full-body massages and all studies where newborns were touched)

Supplementary Table 2. Information on studies included in the meta-analysis and studies that were excluded from the meta-analysis but included in the systematic review. Reasons for exclusion from the meta-analysis comprised either missing data such as means or standard deviations, within-subject designs or studying the effects of touch interventions on animals.

Included studies			Excluded studies		
First author	Year		First author	Year	Reason for exclusion
Ahles	1999		Arditi	2006	Missing data
Albert	2009		Beijers	2016	Within-subject study
Ang	2012		Bigelow	2012	No RCT
Arora	2005		Castral	2008	Missing data
Asadollahi	2016		Charpak	1994	No RCT
Basiri-Moghadam	2015		Cho	2016	No RCT
Bauer	2010		Choi	2015	No RCT
Bennett	2015		Choudhary	2016	Within-subject study
Bergamm	2007		Cloutier	2015	Animal study
Berretz	2021		Cloutier	2014	Animal study
Billhult	2007		Cloutier	2008	Animal study
Billhult	2008a		Cong	2012	Within-subject study
Billhult	2008b		Cong	2011	Within-subject study
Braun	2012		Cong	2009	Missing data
Cabibhan	2017		Costa	2020	Animal study
Campeau	2007		Coulon	2015	Animal study
Can	2021		Diego	2007	Missing data
Carfoot	2005		Diego	2005	Missing data
Cattaneo	2007		El-Farrash	2020	Missing data
Christensson	1992		Escalona	2001	Missing data
Cutshall	2010		Feldman	2002	No RCT
Dalili	2016		Feldman	2003	No RCT

Diego	2001
Diego	2002
Diego	2002
Diego	2005
Diego	2007
Diego	2008
Dieter	2003
Ditzen	2007
Dreisoerner	2021
Eaton	1986
Edens	1992
El-Farrash	2020
Erlandsson	2007
Fattah	2011
Feldman	2010
Field	1996
Field	1997b
Field	2009b
Field	1986
Field	1997a
Field	2002
Field	2003
Field	2007
Field	2008
Field	2009a
Field	2011
Field	2014
Filho	2015
Forward	2015
Fraser	1993
Frey-Law	2008
Gao	2015
Gathwala	2010
Geva	2020
Givi	2013
Goldstein-Ferber	2002
Goldstein-Ferber	2004
Gomes	2009
Gonzalez	2009
Gray	2000
Grewen	2003
Gürol	2010
Haley	2012

Feldman	2007	No RCT
Ferreira	2010	No RCT
Fidanza	2021	Within-subject study
Field	2001	Missing data
Field	2007	Missing data
Field	2016	Missing data
Field	2004	Missing data
Field	1998	Missing data
Field	1997	Missing data
Field	1999	Missing data
Field	1992	Missing data
Field	1998	Missing data
Field	1997	Missing data
Garner	2008	No RCT
Gitau	2002	Missing data
Glover	2002	Missing data
Gourkow	2014	Animal study
Groer	2002	Animal study
Haley	2012	Missing data
Harris	2012	Missing data
Hart	2001	Missing data
Henricson	2008	Missing data
Hinchcliffe	2020	Animal study
Hodgson	2008	Within-subject study
Holst	2005	Animal study
Hori	2014	Animal study
Hori	2013	Animal study
Hucklenbruch-Rother	2020	Missing data
Jain	2006	Within-subject study
Johnston	2003	Within-subject study
Johnston	2008	Within-subject study
Jung	2006	Missing data
Karagozoglu	2013	No RCT
Karbasi	2013	No RCT
Kim	2001	No RCT
Kim	2017	No RCT
Koole	2014	Missing data
Kuhn	1991	Missing data
Lee	2006	No RCT
Lindgren	2010	Within-subject study
Lund	2006	Missing data
Ma	2022	Animal study
Matsunaga	2009	Within-subject study

Hattan	2002
Haynes	2022
Hernandez-Reif	2001b
Hernandez-Reif	1998a
Hernandez-Reif	1998b
Hernandez-Reif	1999
Hernandez-Reif	2000
Hernandez-Reif	2001a
Hernandez-Reif	2002
Hernandez-Reif	2004
Hernandez-Reif	2007
Hertenstein	2009
Hoffmann	2021
Im	2009
Kapoor	2017
Kashaninia	2008
Kelling	2016
Khilnani	2003
Kianmehr	2014
Kim	2016a
Krohn	2011
Kumar	2013
Leivadi	1999
Lin	2015
Lindgren	2013
Listing	2009
Ludington-Hoe	2004
Luong	2016
Massaro	2009
Mathai	2001
Mendes	2008
Mirnia	2017
Mitchinson	2007
Modrcin-Talbott	2003
Mok	2004
Mokaberian	2021
Moyer-Mileur	2013
Moyle	2014
Negahban	2013
Nelson	1986
Nunes	2016
O'Higgins	2008
Oliveira	2012

Mitchell	2013	Missing data
Mori	2004	Within-subject study
Muntsant	2019	Animal study
Ohgi	2002	No RCT
Okan	2010	Missing data
Olsson	2016	Within-subject study
Pauk	1986	Animal study
Reddan	2020	Missing data
Rose	1980	Missing data
Scafidi	1990	No RCT
Seyyedrasooli	2014	Missing data
Sharpe	2007	Missing data
Shor-Posner	2006	Missing data
Simpson	2019	Animal study
Simpson	2019	Animal study
Smith	2013	Missing data
Smith	2013	Missing data
Soares	2011	Animal study
Solkoff	1975	Missing data
Stringer	2008	Missing data
Walach	2003	Missing data
Walker	2022	Animal study
Woods	2009	Missing data
Yamaguchi	2020	Within-subject study
Yates	2014	Within-subject study
Yu	2022	Animal study

Pinazo	2020			
Pope	1994			
Preyde	2000			
Ramanathan	2001			
Rodriguez-Mansilla	2014			
Scafidi	1986			
Scafidi	1996			
Scarr-salapatek	1972			
Serrano	2016			
Sherman	2009			
Shiloh	2003			
Srivastava	2014			
Sui-Whi	2011			
Suman	2008			
Sumioka	2013			
Sumioka	2021			
Suzuki	2010			
Thomson	2011			
Triplett	1979			
Weinrich	1990			
Wheeden	1993			
White	1976			
Wilkie	2000			
Willemse	2017			
Willemse	2019			
Yamazaki	2016			
Yang	2015			

Supplementary Table 3. Information on all studies excluded and which inclusion criterion was not met leading to exclusion of the study. Note that studies could fulfill multiple exclusion criteria simultaneously.

First author	Year	Paper Type	Inclusion criteria 1: Health outcome measurement (1 = yes, 0 = no)	Inclusion criteria 2: Touch as part of an intervention (1 = yes, 0 = no)	Inclusion criteria 3: The experimental group is distinguished to the control group through touch alone (1 = yes, 0 = no)
Acolet	1993	Quantitative Study	1	1	0
Acolet	1989	Quantitative Study	1	1	0
Adam	2002	Quantitative Study	1	1	0
Adam	2010	Mixed Study	1	1	0
Adamson-Macedo	1985	Quantitative Study	1	1	0

Affonso	1993	Quantitative Study	0	0	0
Aghabati	2008	Quantitative Study	1	1	0
Ahmed	2011	Quantitative Study	1	1	0
Airosa	2016	Mixed Study	1	1	0
Alander	2015	Qualitative Study	1	0	0
Allen	1991	Quantitative Study	1	0	1
Allen	2002	Quantitative Study	1	0	1
Ander	2011	Qualitative Study	1	1	0
Ander	2013	Qualitative Study	1	1	0
Anderson	1992	Quantitative Study	1	0	1
Backus	2016	Pilot Study	1	1	0
Bailie	2015	Quantitative Study	1	0	1
Banks	2008	Quantitative Study	1	0	1
Basler	2011	Pilot Study	1	1	0
Batson	1998	Quantitative Study	1	0	0
Bauer	1997	Quantitative Study	1	1	0
Bauer	1996	Quantitative Study	1	1	0
Bauer	1987	No PDF	-	-	-
Baun	1991	Quantitative Study	0	0	0
Baun	1984	Quantitative Study	1	1	0
Begum	2008	Quantitative Study	1	1	0
Belgrave	2009	Quantitative Study	0	0	0
Bera	2014	Quantitative Study	1	1	0
Bernstein	2000	Qualitative study	1	1	0
Berry	2012	Pilot Study	1	1	0
Beth	2006	Pilot Study	1	1	0
Bingley	2007	Quantitative Study	1	0	0
Bisiani	2012	Case Study	1	1	0
Bjorling	2019	Qualitative study	1	1	0
Blankfield	2001	Quantitative Study	0	0	0
Bohm	1997	Quantitative Study	0	0	0
Bohnhorst	2001	Quantitative Study	1	1	0
Boitor	2015	Pilot Study	1	1	0
Boyd	2016	Meta-Analysis	-	-	-
Boyd	2016	Meta-Analysis	-	-	-

Brauer	2016	Quantitative Study	0	0	0
Brett	2018	Quantitative Study	1	1	0
Brett	2018	Quantitative Study	1	1	0
Brooker	2011	Case Study	0	0	0
Browne	2018	Quantitative Study	1	1	0
Budge	1998	Quantitative Study	1	0	1
Burgdorf	2001	Quantitative Study	1	1	0
Burkett	2016	Quantitative Study	0	1	1
Burleson	2021	Quantitative Study	1	0	0
Bush	2012	Quantitative Study	0	0	0
Buttagat	2012	Quantitative Study	1	1	0
Cady	1997	Quantitative Study	1	1	0
Camic	2017	Quantitative Study	1	1	0
Casler	1965	Quantitative Study	0	0	0
Cassileth	2004	Quantitative Study	1	0	0
Chatel-Goldman	2014	Qualitative Study	0	0	0
Chen	2007	Qualitative Study	1	1	0
Chen	2020	Quantitative Study	1	1	0
Chen	2015	Quantitative Study	1	1	0
Cheng	2013	Meta-Analysis	-	-	-
Cheung	2017	quantitative study	1	1	0
Chow	2020	Case Study	1	0	1
Christensson	1995	Quantitative Study	1	1	0
Chu	2009	No PDF	-	-	-
Churchill	1999	No PDF	-	-	-
Coan	2006	Quantitative Study	0	0	0
Cohen	2014	Quantitative Study	1	0	0
Cole	1995	Case study	1	1	0
Colombo	2006	Quantitative Study	1	0	1
Cook	2004	Quantitative Study	0	0	0
Cordes	2017	Quantitative Study	1	1	0
Corner	1995	Quantitative Study	1	1	0
Crane	2012	Quantitative Study	1	1	0
Crawford	2016	Meta-Analysis	-	-	-

Croy	2016	Quantitative Study	0	0	0
Croy	2019	Quantitative Study	0	0	0
Cullen	1999	Review	-	-	-
Daga	1998	Quantitative Study	1	1	0
Dalbye	2011	Quantitative Study	0	0	0
Debrot	2013	Quantitative Study	1	0	0
Debrot	2013	Quantitative Study	1	0	0
Delgado	2018	Quantitative Study	1	1	0
Della Longa	2021	Quantitative Study	1	1	0
Dembicki	1996	Quantitative Study	1	0	1
Denison	2004	Quantitative Study	0	0	0
Dickstein	2004	Quantitative Study	0	1	1
Dickstein	2009	Quantitative Study	0	1	1
Diego	2004	Quantitative Study	1	1	0
Diego	2009	Quantitative Study	1	1	0
Dion	2016	Pilot Study	1	1	0
Donoyama	2012	Case Study	1	0	0
Donoyama	2010	Quantitative Study	1	1	0
Drapeau	2012	Quantitative Study	0	0	0
Drescher	1985	Quantitative Study	1	1	0
Earles	2015	Quantitative Study	1	1	0
Egmose	2018	Quantitative Study	1	0	0
Ejindu	2007	Pilot Study	1	1	0
Elder	2017	Quantitative Study	1	1	0
Ellingford	2007	No PDF	-	-	-
Ellingsen	2014	Quantitative Study	1	1	0
Elliott	2012	Quantitative Study	1	1	0
Enders	2013	Quantitative Study	0	0	1
Erk	2015	Quantitative Study	0	0	0
Esposito	2013	Quantitative Study	1	1	0
Esteves	2015	Case Study	1	1	0
Fairhurst	2014	Quantitative Study	1	1	0
Fallah	2013	Quantitative Study	1	1	0
Fatollahzade	2020	Quantitative Study	1	1	0

Feldman	2010	Quantitative Study	0	0	0
Feldman	2010	Quantitative Study	0	0	0
Ferber	2002	Quantitative Study	1	1	0
Ferrell-Torry	1993	Quantitative Study	1	1	0
Fick	1993	Quantitative Study	1	1	0
Field	1998	Quantitative Study	1	1	0
Field	1997	No PDF	-	-	-
Field	2002	Review	-	-	-
Field	1996	Quantitative Study	1	1	0
Field	2006	Quantitative Study	1	1	0
Field	1996	Quantitative Study	1	1	0
Field	1998	Quantitative Study	1	1	0
Field	1996	Quantitative Study	1	1	0
Field	2013	Quantitative Study	1	1	0
Field	2004	Quantitative Study	1	1	0
Field	2006	Quantitative Study	0	1	1
Field	2005	Review	-	-	-
Field	1996	Quantitative Study	1	1	0
Field	1998	Quantitative Study	1	0	0
Field	1998	No PDF	-	-	-
Field	1997	Quantitative Study	1	1	0
Fisher	1976	Quantitative Study	0	0	0
Floyd	2009	Quantitative Study	1	1	0
Fogg	1998	Qualitative study	0	1	0
Franzén	2011	Quantitative Study	0	0	1
Franzén	2012	Quantitative Study	0	0	1
Friedmann	1980	Quantitative Study	1	0	1
Friedmann	1983	Quantitative Study	1	0	1
Gagne	1994	Quantitative Study	1	0	0
Gagnon	2004	Quantitative Study	1	1	0
Garrity	1989	Quantitative Study	1	0	0
Gazzolo	2000	Quantitative Study	1	1	0
Gee	2015	Quantitative Study	1	1	0

Goodall	2019	Quantitative Study	1	0	1
Gordon	1998	Quantitative Study	1	0	1
Gordon	2006	Quantitative Study	1	1	0
Grandi	2015	Quantitative Study	1	1	0
Grealish	2000	Quantitative Study	1	1	0
Green	2011	Quantitative Study	0	1	1
Grewen	2005	Quantitative Study	1	0	0
Grunwald	2014	Quantitative Study	0	0	0
Guzetta	2011	Quantitative Study	0	1	1
Haller	2016	Quantitative Study	1	1	0
Han	2019	Qualitative Study	1	1	0
Hänsel	2011	Quantitative Study	1	0	0
Harrison	1990	Quantitative Study	1	1	0
Harrison	1996	Quantitative Study	1	1	0
Hatayama	2008	Quantitative Study	1	1	0
Havener	2009	Pilot Study	1	0	1
Hawkins	2021	Qualitative Study	1	0	1
Hawranik	2008	Quantitative Study	1	0	0
Henricson	2008	Quantitative Study	0	1	1
Hernandez-Reif	1998	No PDF	-	-	-
Hernandez-Reif		No PDF	-	-	-
Herrald	2006	Quantitative Study	1	0	1
Hicks-moore	2008	Quantitative Study	0	1	1
Ho	2010	Quantitative Study	1	1	0
Ho	2010	Quantitative Study	1	1	0
Holliday-Welsh	2009	Quantitative Study	1	1	0
Hovind	1974	No PDF	-	-	-
Huff-Mercer	2015	Qualitative Study	1	1	0
Iida	2000	Quantitative Study	1	1	0
Im	2009	Quantitative Study	1	1	0
Ingersoll	1994	Quantitative Study	1	0	1
Insana	2008	Quantitative Study	0	1	1
Ironson	1996	Quantitative Study	1	1	0

Jabraeile	2016	Quantitative Study	1	1	0
Jakubiak	2016	Quantitative Study	1	0	0
James	2006	Quantitative Study	1	1	0
Jay	1982	Dissertation	-	-	-
Jinon	1996	No PDF	-	-	-
Johannsen	2010	Quantitative Study	0	1	1
Johnson	1989	Quantitative Study	1	0	1
Johnston	2009	Quantitative Study	1	1	0
Johnston	2011	Quantitative Study	1	1	0
Jones	1999	Quantitative Study	0	1	1
Jones	1998	Quantitative Study	0	1	1
Jönsson	2018	Quantitative Study	0	0	0
Jung	2017	Quantitative Study	1	1	0
Kamali	2014	Quantitative Study	1	1	0
Kanamori	2001	Case Study	1	0	1
Kanamori	2002	Case Study	1	1	0
Karlsson	2022	Qualitative Study	1	1	0
Karlsson	2012	Quantitative Study	1	1	0
Kawamura	2007	Quantitative Study	1	1	0
Kelly	2017	Quantitative Study	0	0	0
Kennedy	2018	Mixed Study	1	1	0
Khun	1990	Quantitative Study	1	0	1
Kim	1999	Quantitative Study	1	1	0
Kimata	2006	Quantitative Study	1	1	0
Kimata	2003	Quantitative Study	1	1	0
Kimura	2010	Quantitative Study	1	1	0
Kloep	2016	Qualitative Study	1	1	0
Ko	2016	Quantitative Study	1	0	1
Kodesh	2015	Quantitative Study	0	1	1
Kommers	2017	Quantitative Study	1	1	0
Kommers	2018	Quantitative Study	0	1	1
Kong	2012	Meta-analysis	-	-	-
Kongable	1989	Quantitative Study	1	1	0
Kotrschal	2015	Quantitative Study	1	1	0

Kovacs	2003	Qualitative Study	1	1	0
Kovacs	2006	Case study	1	1	0
Krahé	2016	Quantitative Study	1	1	0
Kramer	1975	No PDF	-	-	-
Kramer	2009	Quantitative Study	1	1	0
Kubsch	2001	Quantitative Study	1	1	0
Kukimoto	2017	Meta-analysis	-	-	-
Kutner	2008	Quantitative Study	1	1	0
Kutner	2008	Quantitative Study	1	1	0
Kutner	2008	Quantitative Study	1	1	0
Lahat	2007	Quantitative Study	1	1	0
Lanceley	2011	Qualitative Study	1	1	0
Lechner	2007	Quantitative Study	1	1	0
Lee	2020	Quantitative Study	1	0	0
Lee	2005	Quantitative Study	1	1	0
Lee	2006	Qualitative study	0	1	1
Lee	2018	Quantitative Study	0	1	1
Lee	2021	Quantitative Study	0	1	1
Lee Zasloff	1994	Quantitative Study	1	0	1
Li	2014	Meta-analysis	-	-	-
Li	2018	Quantitative Study	0	1	1
Libin	2004	Pilot study	1	1	0
Lida	2000	Quantitative Study	1	1	0
Light	2005	Quantitative Study	1	1	0
Liljencrantz	2017	Quantitative Study	1	1	0
Lindgren	2012	Quantitative Study	0	1	0
Longworth	1982	Quantitative Study	1	1	0
Lovas	2002	Pilot Study	0	1	1
Ludington-Hoe	1991	Quantitative Study	0	1	1
Ludington-Hoe	1999	Quantitative Study	1	1	0
Lutgendorf	2010	Quantitative Study	1	0	1
Mackenzie	2006	Pilot Study	1	1	0
Mackness	2021	Quantitative Study	1	0	0
Majic	2013	Quantitative Study	1	0	0

Mällo	2007	Quantitative Study	0	1	0
Manzotti	2020	Quantitative Study	1	1	0
Manzotti	2019	Quantitative Study	1	1	0
Maratos	2017	Quantitative Study	1	1	0
Maratos	2017	Quantitative Study	1	1	0
Marcia	2008	Qualitative Study	0	1	1
Martin	1998	Book	0	0	0
Martin	2002	Quantitative Study	1	1	0
Maseda	2018	Quantitative Study	1	0	0
Matourpour	2015	Quantitative Study	1	0	0
Maville	2008	Pilot Study	1	1	0
McCabe	2002	Quantitative Study	1	1	0
McCain	2005	Case Study	1	1	0
McKechnie	1983	Quantitative Study	1	1	0
Meek	1993	Quantitative Study	1	1	0
Minsbull	2009	No PDF	-	-	-
Mojtabai	2011	Quantitative Study	0	0	0
Mojtabai	2011	Quantitative Study	0	0	0
Moore	2012	Meta-analysis	-	-	-
Moraska	2016	Pilot Study	1	1	0
Moraska	2008	Pilot Study	1	1	0
Morelius	2005	Quantitative Study	1	1	0
Morelius	2005	Quantitative Study	1	1	0
Morhenn	2008	Quantitative Study	0	1	1
Morse	2021	Mixed Study	1	1	0
Mossello	2011	Quantitative Study	1	1	0
Motomura	2004	Quantitative Study	1	1	0
Motomura	2004	Quantitative Study	1	1	0
Mourey	2017	Quantitative Study	1	0	1
Moyer	2004	Meta-analysis	-	-	-
Moyle	2011	Quantitative Study	1	1	0
Murphy	2018	Quantitative Study	1	1	0
Nakajima	2001	Quantitative Study	1	1	0
Naruse	2021	Qualitative Study	1	1	0
Neu	2009	quantitative study	1	1	0

Nicholls	2013	Qualitative Study	1	1	0
Norberg	1986	Case Study	0	1	1
Nordgren	2012	Pilot Study	1	1	0
Noto	2010	Quantitative Study	1	1	0
Noto	2010	Quantitative Study	1	1	0
Noto	2010	Quantitative Study	1	1	0
Novak	2021	Quantitative Study	1	1	0
O'Haire	2013	Quantitative Study	0	1	1
Oliveira	2018	Quantitative Study	1	1	0
Ostermann	2008	Quantitative Study	1	1	0
Packheiser	2021	Quantitative Study	1	1	0
Parslow	2015	Quantitative Study	1	0	1
Parslow	2005	Quantitative Study	1	0	1
Pawling	2017	Quantitative Study	1	1	0
Pedersen	2011	Quantitative Study	1	1	0
Peláez-Nogueras	1996	Quantitative Study	1	1	0
Peled-Avron	2018	Quantitative Study	0	1	1
Pezzati	2014	Quantitative Study	0	1	1
Pichel	1989	case study	1	1	0
Pickles	2016	Quantitative Study	1	0	0
Pitheckoff	2016	Qualitative Study	1	1	0
Poland	2002	No PDF	-	-	-
Post-White	2009	Pilot Study	1	1	0
Post-White	2003	Quantitative Study	1	0	1
Procianoy	2010	Quantitative Study	1	1	0
Pu	2019	Qualitative study	1	1	0
Raina	1999	Quantitative Study	1	0	1
Rangey	2014	Quantitative Study	1	1	0
Ranjbaran	2017	Meta-analysis	-	-	-
Rapaport	2012	Quantitative Study	1	1	0
Rapaport	1998	Quantitative Study	1	1	0
Rault	2019	Quantitative Study	0	1	1
Remington	2002	Quantitative Study	0	0	0
Rexilius	2002	Quantitative Study	1	0	1

Rice	1979	Quantitative Study	-	-	-
Richeson	2003	Pilot Study	1	1	0
Riquelme	2016	Quantitative Study	0	0	1
Roberts	2022	Quantitative Study	1	1	0
Robinson	2015	Quantitative Study	1	1	0
Robison	2016	Quantitative Study	1	1	0
Rodrigues	2004	Quantitative Study	0	1	1
Routasalo	1996	Quantitative Study	1	0	1
Rudnicki	2012	Quantitative Study	1	1	0
Sabanovic	2013	Qualitative Study	1	1	0
Sailer	2022	Quantitative Study	0	0	0
Sailer	2019	Quantitative Study	0	0	0
Sams	2006	Pilot Study	0	1	1
Sansone	2000	Quantitative Study	1	1	0
Saxton	2015	Quantitative Study	1	0	1
Scheele	2014	Quantitative Study	0	1	1
Schino	1988	Quantitative Study	0	1	1
Scott	1979	Quantitative Study	1	1	0
Scott	1983	Quantitative Study	1	1	0
Sefidgar	2016	Quantitative Study	1	1	0
Sehlsedt	2016	Quantitative Study	0	0	0
Sellers	2005	Qualitative Study	1	0	0
Serin	2018	Quantitative Study	1	1	0
Serpell	1991	Pilot Study	1	0	1
Seyyedrasooli	2017	No PDF	-	-	-
Sharp	2012	Quantitative Study	1	0	0
Sheidaei	2016	Quantitative Study	1	1	0
Shin	2014	Quantitative Study	1	1	0
Shiomi	2017	Quantitative Study	0	1	1
Shiomi	2018	quantitative Study	1	1	0
Shoemaker	1997	Quantitative Study	1	1	0
Siegel	1990	Quantitative Study	1	0	1
Siegel	1999	Quantitative Study	1	0	1

Simington	1993	Quantitative Study	1	0	1
Sims	1986	No abstract	-	-	-
skovdahl	2007	Qualitative Study	1	1	0
Smiraglia	2015	Quantitative Study	1	1	0
Sobo	2006	Pilot Study	1	1	0
Sockalingam	2008	Case study	1	1	0
Soler	2015	Quantitative Study	1	1	0
Stasi	2004	Quantitative Study	1	1	0
Stiehl	2005	Other	1	1	0
Strauss	2018	Quantitative Study	1	1	0
Suchecki	1993	Quantitative Study	1	1	0
Suresh	2008	Quantitative Study	1	1	0
Synder	1995	Quantitative Study	1	1	0
Tabatabaee	2016	Quantitative Study	1	0	1
Tai	2011	Quantitative Study	1	1	0
Takahashi	2011	Quantitative Study	1	1	0
Takeuchi	2010	Quantitative Study	1	0	0
Tamura	2004	Quantitative Study	1	0	1
Tamura	2001	Quantitative Study	1	1	0
Tessier	2003	Quantitative Study	0	0	0
Theofanopoulou	2019	Quantitative Study	0	1	1
Thodberg	2016	Quantitative Study	0	1	1
Thomson	2014	Mixed Study	1	1	0
Tribet	2008	Qualitative Study	1	1	0
Triscoli	2019	Quantitative Study	1	0	0
Triscoli	2017	Quantitative Study	1	1	0
Trotter	2018	Quantitative Study	0	0	0
Tsuji	2015	Pilot Study	1	1	0
Tuulari	2019	Quantitative Study	0	0	0
Tyler	1990	Quantitative Study	1	1	0
Ünal Aslan	2021	Quantitative Study	1	0	0
van Puyvelde	2019	Quantitative Study	1	1	0
van Puyvelde	2019	Quantitative Study	1	1	0
van Raalte	2021	Quantitative Study	1	1	0

Vannorsdall	2004	Quantitative Study	1	1	0
Varela	2018	Quantitative Study	1	1	0
Velandia	2010	Quantitative Study	1	1	0
Viau	2010	Quantitative Study	1	1	0
Vittner	2019	Quantitative Study	1	0	0
von Mohr	2021	Quantitative Study	1	0	0
von Mohr	2017	Quantitative Study	0	0	0
von Mohr	2018	Quantitative Study	0	1	0
Vormbrock	1988	Quantitative Study	1	1	0
Vural Dogru		Quantitative Study	1	0	0
Wada	2005	Qualitative Study	1	1	0
Wada	2007	Quantitative Study	1	1	0
Wada	2008	Quantitative Study	1	1	0
Walsh	1995	Quantitative Study	1	1	0
Wang	2006	Quantitative Study	1	0	0
Wang	2013	Meta-analysis	-	-	-
Wang	2019	Quantitative Study	0	0	0
Wang	2020	Quantitative Study	0	1	1
Wardell	2008	Qualitative Study	1	1	0
Weiss	2001	Quantitative Study	1	1	0
Weiss	2009	Quantitative Study	1	0	0
Weiss	1990	Quantitative Study	1	1	0
Weze	2007	Quantitative Study	1	0	0
Weze	2004	Quantitative Study	1	1	0
Whitcher	1979	Quantitative Study	0	1	1
Wikström	2003	Pilot Study	1	1	0
Wilhelm	2001	Quantitative Study	1	1	0
Winkler	1989	Quantitative Study	1	1	0
Witucki	1997	Quantitative Study	1	1	0
Woods	2005	Quantitative Study	1	0	0
Woods	2002	Quantitative Study	1	0	0
Woytuk	2020	Quantitative Study	0	0	0
Wu	2017	Meta-analysis	-	-	-

Wu	2002	Quantitative Study	1	1	0
Yoshida	2020	Quantitative Study	1	1	0
Young	2020	Qualitative Study	1	1	0
Youssef	2013	Quantitative Study	1	1	0
Yu	2015	Quantitative Study	-	-	-
Yu	2015	Quantitative Study	0	1	1
Yuan	2015	Meta-analysis	-	-	-
Zhang	2012	Quantitative Study	1	1	0
Zisselman	1996	Quantitative Study	1	1	0

Supplementary Table 4. Moderator description and coding specificities.

Moderator	Description	(Re)-Coding of variables
Health outcome (categorical)	On a broader scale, we used a dichotomous classification system (mental or physical health outcomes). To provide more detailed insight, we extracted health outcomes specifically as well. In newborns, mental health-like outcomes were rarely described as they are difficult to determine. These outcomes were assessed through behavioral means such as crying or parental assessment of mood.	All health outcomes were either classified as “Mental Health” (e.g., state anxiety, depression, or positive affect) or “Physical Health” outcomes (e.g., cortisol, pain or respiration). As specific health outcomes were highly diverse, we also re-coded them to form larger sub-groups. For example, back pain, stretch pain or headache were subsumed in a larger category of “pain”. Anxiety was divided into state and trait anxiety depending if the study inquired about momentary/state anxiety (e.g., through the STAI-S[49]) or longer-lasting symptoms of anxiety (at least over the past seven days through for example the STAI-T).
Touch dyad (categorical)	Touch dyads were defined as human-human touch (humans being touched by other humans), human-robot touch (humans being touched by robots), human-object touch (humans touching objects) and human-animal touch (humans touch animals). This moderator was not investigated in the newborns meta-analysis since no study used object/robot/animal touch.	For children/adults, we re-coded this variable into “Human-Human” (all studies involving humans as toucher and touched individual) and “Human-Object” (all studies involving robots or objects touching or being touched by humans). Only a single study measured the effects of animal touch. We therefore excluded this study from moderator analysis in this category.
Skin-to-skin contact (categorical)	For all studies, we extracted if the touch application involved skin-to-skin contact between the touching dyad. This moderator was not analyzed in the newborn meta-analysis as there was only a single study without skin-to-skin contact.	Studies were coded as skin-to-skin contact if the head, neck or hands were touched or if massage oils were used. By default, studies involving human-robot or human-object touch did not use skin-to-skin contact.
Type of touch (categorical)	We assessed the type of touch that was applied in a given situation. The most	Since massage therapy was the prime interest of previous studies as a touch

	prevalent type of touch was massage therapy for adults or kangaroo care in case of newborns. Other types of touch included for example hugs, gentle touch, tactile-kinesthetic stimulation or hand-holding.	intervention, we were interested in whether massages provide stronger benefits compared to other forms of touch. Since other touch forms were highly diverse and thus rarely found across multiple studies for children/adults, we re-coded this moderator dichotomously in "Massage Therapy" and "Other Touch Types". Since a large number of studies used "Kangaroo Care" in newborns, we included this type of touch as an additional factor level in the newborns meta-analysis.
Clinical status (categorical)	Cohorts were both dichotomously divided into clinical and healthy individuals or the underlying disorder was specified.	Specific disorder types were re-coded into "Cancer" patients (e.g., breast cancer or bone cancer), patients with "Neurological Disorders" (e.g., Parkinson or dementia), patients with "Pain Disorders" (e.g., chronic back pain or fibromyalgia), patients with "Depression Disorders" (e.g., postpartum depression or major depressive disorder) or patients undergoing "Surgery" (e.g., aortic surgery or hip replacement). Patients with other kinds of disorders were subsumed in the "Other Conditions" category. For newborns, no re-classification was performed as the only clinical condition comprised premature birth with the exception of a single study investigating neonatal jaundice[50].
Touched body part (categorical)	We recorded the body part (arm, leg, head or back) that was touched for moderation.	If more than one region was touched during the intervention, we classified it as "multiple regions". This moderator was omitted for the newborns meta-analysis as all studies used multi-regional touch.
Familiarity of the touch dyad (categorical)	Familiarity of the dyad was coded dichotomously as familiar or unfamiliar.	In case spouses, friends or parents were touching each other, we recorded the intervention as familiar touch. Otherwise, for example if touch was applied by the experimenter or health care personnel, the moderator was classified as unfamiliar.
Familiarity of the location (categorical)	Familiarity of the location was coded dichotomously as familiar or unfamiliar.	Interventions conducted in laboratories, hospitals or massage studios were classified as unfamiliar. Interventions at the participants' homes were classified as familiar. For newborns, we omitted this moderator as there were no truly familiar locations in that case

Sex of touched individual (continuous)	To account for potential sex differences, we assessed the sex of the person receiving the touch in a given cohort.	Since only seven effects were found for male child/adult cohorts and a large number of studies used mixed cohorts, we decided to convert this moderator from categorical to a continuous one using the ratio between sampled women/girls and men/boys. If a cohort comprised only women, the ratio was set to 100 whereas it was set to 0 if only men were sampled.
Number of sessions (continuous)	We assessed how often the touch intervention occurred prior to measurement of the outcome.	Number of sessions was measured as a simple frequency of the touch interventions.
Duration of touch (continuous)	We determined how long the touch was applied per session.	Duration was measured in minutes. Total duration (session duration * session number) was not investigated as a moderator as it was very strongly correlated with session number.
Mean age (continuous)	Mean age was used as predictor of potential age differences.	Mean age was computed across the experimental and control group for a given effect. Since age did not vary in newborns, this moderator was excluded from the newborns meta-analysis.
Directionality (categorical)	Studies were either coded as unidirectional or bi-directional touch.	Studies were classified as unidirectional whenever one person was a clear receiver and one person was a clear provider of touch. Studies were classified as bi-directional if touch was applied and received by both individuals in the dyad. This moderator was not investigated in newborns since touch interventions are never truly bi-directional in this case. If the health outcome was measured in the parent during kangaroo care, it was coded as bi-directional.
Study location (categorical)	We documented the region in which a study was conducted.	Study location was coded into North America, South America, Asia, Europe, Africa or Oceania. If a study explicitly mentioned for example a university or hospital as the location of study, we used this measure. In case there was no mention, the affiliation of the first author of the study was used instead.

Supplementary Table 5. Alphabetized list of final outcomes separate for newborns and adults. The table is separated by outcomes with sufficient power for further analysis and outcomes for which sufficient effects could not be extracted to allow for further analysis. Outcomes are colorized as physical health (PH) effects in magenta and mental health (MH) effects in blue. The number of available effects per outcome is listed in brackets. NA = not categorized.

Outcomes for newborns that were analyzed at the specific outcome level ($n \geq 8$ effects)	Outcomes for adults that were analyzed at the specific outcome level ($n \geq 9$ effects)
Bowel movements (10, PH)	Anxiety, state (78, MH)
Cortisol (8, PH)	Anxiety, trait (25, MH)
Heart rate (12, PH)	Depression, trait (33, MH)
Liver enzymes (9, PH)	Diastolic/systolic blood pressure (21 each, PH)
Respiration (12, PH)	Fatigue (10, PH)
Temperature (9, PH)	Heart rate (31, PH)
Weight (45, PH)	Mobility (10, PH)
	Negative affect (33, MH)
	Pain (65, PH)
	Positive affect (18, MH)
	Respiration (13, PH)
	Sleep (14, PH)

Outcomes for newborns that were not analyzed at the specific outcome level ($n < 8$ effects)	Outcomes for adults that were not analyzed at the specific outcome level ($n < 9$ effects)
Affect regulation (5, MH)	Affect regulation (1, MH)
Arousal (3, MH)	Anxiety, state/trait (3, MH)
Autonomic stability (3, PH)	Arousal (7, MH)
Bacterial decolonization (1, PH)	Autism behavior (1, MH)
Blood sugar (1, PH)	Balance (2, PH)
Death rate (3, PH)	Blood sugar (1, PH)
Depression, trait (1, MH)	Body satisfaction (1, MH)
Food intake (7, PH)	Disability (2, PH)

Grimacing (2, NA)	Disease assessment (1, PH)
Hospital stay (7, PH)	Emotional function (1, MH)
Immune system (2, PH)	Food intake (5, PH)
Motor function (4, PH)	Hospital stay (1, PH)
Negative affect, state (5, MH)	Immune system (5, PH)
Oxygen saturation (5, PH)	Insulin (1, PH)
Pain (3, PH)	Intellectual function (1, MH)
pH levels (1, PH)	Motor function (1, PH)
Postnatal complications (1, PH)	Muscle strength (1, PH)
Sleep (7, PH)	Nausea (4, PH)
Stress (7, MH)	Oxygen saturation (1, PH)
Urinary bone metabolism (1, PH)	Relaxation (6, MH)
	Social function (3, MH)
	Somatic/vegetative state (1, PH)
	Somatization (1, PH)
	Stereotypical behavior (1, MH)
	Stress (7, MH)
	Well-being, trait (5, MH)
